# Compartment-specific small non-coding RNA changes and nucleolar defects in human mesial temporal lobe epilepsy

**DOI:** 10.1007/s00401-024-02817-8

**Published:** 2024-11-07

**Authors:** Vamshidhar R. Vangoor, Giuliano Giuliani, Marina de Wit, Carolina K. Rangel, Morten T. Venø, Joran T. Schulte, Andreia Gomes-Duarte, Ketharini Senthilkumar, Noora Puhakka, Jørgen Kjems, Pierre N. E. de Graan, R. Jeroen Pasterkamp

**Affiliations:** 1 Department of Translational Neuroscience, University Medical Center Utrecht Brain Center, University Medical Center Utrecht, Utrecht University, 3584 CG Utrecht, The Netherlands; 2 Interdisciplinary Nanoscience Centre, Department of Molecular Biology and Genetics, Aarhus University, 8000 Aarhus, Denmark; 3 Omiics ApS, 8200 Aarhus N, Denmark; 4 Princess Máxima Center for Pediatric Oncology, Heidelberglaan 25, 3584 CS Utrecht, The Netherlands; 5 VectorY Therapeutics, Matrix Innovation Center VI, Science Park 408, 1098 XH Amsterdam, The Netherlands; 6 A.I. Virtanen Institute for Molecular Sciences, University of Eastern Finland, FI-70211 Kuopio, Finland

**Keywords:** Epilepsy, Human, microRNA, Non-coding RNA, Nucleolus, Small nucleolar RNA

## Abstract

Mesial temporal lobe epilepsy (mTLE) is a debilitating disease characterized by recurrent seizures originating from temporal lobe structures such as the hippocampus. The pathogenic mechanisms underlying mTLE are incompletely understood but include changes in the expression of non-coding RNAs in affected brain regions. Previous work indicates that some of these changes may be selective to specific sub-cellular compartments, but the full extent of these changes and how these sub-cellular compartments themselves are affected remains largely unknown. Here, we performed small RNA sequencing (RNA-seq) of sub-cellular fractions of hippocampal tissue from mTLE patients and controls to determine nuclear and cytoplasmic expression levels of microRNAs (miRNAs). This showed differential expression of miRNAs and isomiRs, several of which displayed enriched nuclear expression in mTLE. Subsequent analysis of *miR-92b*, the most strongly deregulated miRNA in the nucleus, showed accumulation of this miRNA in the nucleolus in mTLE and association with snoRNAs. This prompted us to further study the nucleolus in human mTLE which uncovered several defects, such as altered nucleolar size or shape, mis-localization of nucleolar proteins, and deregulation of snoRNAs, indicative of nucleolar stress. In a rat model of epilepsy, nucleolar phenotypes were detected in the latency period before the onset of spontaneous seizures, suggesting that nucleolar changes may contribute to the development of seizures and mTLE. Overall, these data for the first time implicate nucleolar defects in the pathogenesis of mTLE and provide a valuable framework for further defining the functional consequences of altered sub-cellular RNA profiles in this disease.

**Supplementary Information:**

The online version contains supplementary material available at 10.1007/s00401-024-02817-8.

## Introduction

Epilepsy is one of the most common and severe neurological diseases affecting 70 million individuals worldwide [16, 88]. Mesial temporal lobe epilepsy (mTLE) is the most common form of focal epilepsy where recurrent unprovoked seizures originate from temporal lobe structures in the brain [31]. In addition to treatment with anti-epileptic drugs, surgical resection of epileptic foci is performed in approximately 60% of drug-resistant patients as a last resort to attain seizure freedom [111]. The pathogenic mechanisms underlying mTLE are still incompletely understood but constitute a crucial starting point for the development of further therapeutic strategies.

Transcriptomic studies performed on resected human mTLE patient tissue indicate large-scale deregulation of the gene expression landscape in the brain, highlighting critical pathways underlying inflammation, gliosis, synaptic structure, and neuronal function [26, 41, 42, 44, 55, 90, 106, 114, 130]. Thus far, studies have mostly focused on transcriptomic changes at the tissue or cellular level, but much less is known about sub-cellular gene expression patterns in mTLE. However, it is becoming increasingly evident that it is important to understand the sub-cellular repertoire of different RNA molecules and to understand the mechanisms that govern their distribution within cells [75, 116, 138, 139]. For example, it has been shown that sub-cellular mRNA localization provides a means to spatially control protein production and transport [7, 105, 115]. Further, long non-coding RNA (lncRNA) molecules often exert specific functions in distinct cell compartments, such as the nucleus or cytoplasm [17, 123, 138]. Similarly, studies on small non-coding RNAs (sncRNAs), e.g., microRNAs (miRNAs), identify unexpected localization or functions in the nucleus. MiRNAs are well known for their ability to bind complementary target regions in cognate mRNAs and thereby to function as posttranscriptional regulators by destabilizing target mRNA expression or by inhibiting protein translation in the cytoplasm [6, 29]. However, miRNAs can also act in the nucleus as gene expression regulators, by targeting promoter or enhancer regions, or to control their own biogenesis or that of other miRNAs [40, 48, 50, 117, 129, 140, 142]. Nuclear localization of miRNAs has been described in post-mitotic neuronal cells and in the maintenance of adult neural stem cell quiescence in zebrafish [58, 61]. Our understanding of the neuronal sub-cellular distribution of miRNAs in disease states is rather rudimentary but may aid the dissection of disease mechanisms in brain diseases such as mTLE [56].

Recent studies localize nuclear miRNAs to the nucleolus where these molecules may affect ribosomal RNA (rRNA) biogenesis and protein synthesis (reviewed in [78]. The nucleolus is a nuclear membrane-less substructure of the nucleus in which different ncRNAs localize and function. It serves a canonical function in rRNA synthesis and ribosomal sub-unit assembly required for protein translation [3, 27, 30]. In addition, it can sequester proteins involved in DNA repair and cell cycle progression, thereby regulating their concentration between normal and stress conditions and controlling cellular homeostasis (reviewed in [12, 52, 82, 121]). Conversely, key structural proteins of the nucleolus, e.g., nucleophosmin (NPM1 or B23) and nucleolin (NCL or C23), shuttle between the nucleolus and nucleoplasm or cytoplasm, interact with many protein and RNA molecules, and mediate different functions [52, 71, 113]. The nucleolus senses conditions of cellular stress and mediates the activation of ribosomal machinery for protein production and regulates the localization of proteins required for rescuing cellular processes [21]. Changes in nucleolar morphology and molecular composition affect nucleolar activity and cellular homeostasis, as observed in human diseases such as cancer and neurodegenerative disorders [38, 47, 93, 136]. Small nucleolar RNAs (snoRNAs), a class of regulatory RNAs localized to the nucleolus, regulate rRNA maturation and biogenesis, and the modification of other RNAs with diverse functional roles [13, 37, 63]. However, despite its important physiological functions and implication in disease, the nucleolus remains largely unexplored in the context of mTLE.

Here, we performed small RNA sequencing (RNA-seq) of sub-cellular fractions of hippocampal tissue from mTLE patients and controls to examine nuclear and cytoplasmic expression of miRNAs. This showed differential expression of miRNAs and isomiRs, several of which displayed enriched nuclear expression. More in-depth analysis of *miR-92b*, the most strongly deregulated miRNA in nuclear fractions, showed accumulation of this miRNA in the nucleolus in mTLE and association with snoRNAs in the nucleus. This prompted us to further study the nucleolus which revealed mis-localization of key nucleolar proteins and deregulation of snoRNAs. Overall, these data identify prominent changes in the sub-cellular distribution of miRNAs and other classes of sncRNAs, and for the first time implicate nucleolar dysfunction in mTLE.

## Materials and methods

### Human tissue

Hippocampus (HC) and neocortical (Cx) tissue was obtained from human pharmacoresistant mTLE patients and Cx tissue adjacent to tumor after surgery at the University Medical Center Utrecht, as described previously [56, 124]. Informed consent was obtained from all patients for use of tissue and clinical information for research purposes and all procedures were approved by the Review board of University Medical Center Utrecht (Utrecht, The Netherlands). Postmortem human HC and Cx tissue from Alzheimer’s disease (AD) patients and healthy control individuals was obtained from the Netherlands Brain Bank (Amsterdam, The Netherlands). Written informed consent was obtained from all donors for brain autopsy and for use of the material and clinical information for research purposes, and the study was performed according to the ethical standards set by the 1964 Declaration of Helsinki. Use of postmortem tissue and clinical information for research purposes was approved by the medical ethics board of the Amsterdam University Medical Center (Amsterdam, The Netherlands). For HC, a total of five HC mTLE [non-hippocampal sclerosis (HS) (male and female)] and five control HC (male and female) samples were used for RNA-seq. For Cx, samples from six mTLE [non-HS (male and female)] and six HS International League Against Epilepsy ((ILAE) Type 1 (male and female) or mTLE + HS) patients and six controls were used for RNA-seq. Details of all human tissue samples used for RNA-seq and follow-up experiments are shown in Supplementary excel file (Tab1 and Tab2). All tissue samples were controlled for the presence of anatomical regions by performing Nissl staining on individual cryo-sections.

### Nuclear-cytoplasmic fractionation of human hippocampus and cortex

Before fractionation, frozen brain samples were powdered in dry ice using a mortar pestle. Approximately 500 mg of powdered tissue was resuspended in Hypotonic Lysis Buffer (HLB) [10 mM Tris (pH 7.4), 3 mM CaCl_2_, 2 mM MgCl_2_, 1% Nonidet P-40 (Sigma), protease inhibitor (Catalogue# 11873580001, Roche), 60 U SUPERase-In™ RNase inhibitor/mL (Catalogue# AM2694, ThermoFisher Scientific)] for 10 minutes (min) on ice to lyse: homogenization and lysis were enhanced with the help of 30–80 strokes in a tightly fitting glass Douncer. The cytoplasmic fraction was collected following gentle centrifugation (500 g) for 5 min at 4 °C, and frozen in Qiazol (Qiagen) until RNA purification. The semi-pure nuclear fraction (pellet) was resuspended in 2 ml Sucrose Buffer I [0.32 M Sucrose, 5 mM CaCl_2_, 3 mM Mg(CH_3_COO)_2_, 0.1 mM EDTA, 10 mM Tris (pH 8.0), 0.5% Nonidet P-40, 1 mM DTT, 25 U SUPERase-In/mL] and placed on top of two thicker sucrose cushions: Sucrose Buffer II [1.6 M Sucrose, 3 mM Mg(CH_3_COO)_2_, 0.1 mM EDTA, 10 mM Tris (pH 8.0), 1 mM DTT, Protease Inhibitor, 35 U SUPERase-In/ml], and a Sucrose Buffer III [2.0 M Sucrose, 3 mM Mg(CH_3_COO)_2_, 0.1 mM EDTA, 10 mM Tris (pH 8.0), 1 mM DTT, 35 U SUPERase-In/mL, Protease Inhibitor], respectively, in ultracentrifugation tubes (Beckman Coulter). Samples were centrifuged using a SW41.4 rotor at 30,000 g at 4 °C for 45 min. Pure nuclei were collected and stored in Qiazol (Qiagen) at − 80 °C until RNA extraction was performed.

### RNA extraction and quality check

Total RNA was extracted from fractionated human samples using miRNeasy Mini Kit (Catalogue# 217084, Qiagen) according to the manufacturer's instructions and DNase-I treated using RNase free-DNase (Catalogue# 79254, Qiagen) to eliminate genomic DNA contamination. When required, RNA was further purified using RNA Clean-Up and Concentration Kit (Catalogue# 23600, Norgen Biotek Corporation). Finally, RNA integrity (RIN) value was estimated in an Agilent 2100 Bioanalyzer (Agilent Technologies) (Supplementary excel file (Tab1).

### Quality control fractionation procedure

The efficiency of the fractionation protocol was estimated at the protein level by western blotting (WB) and at the RNA level by quantitative PCR (RT-qPCR). For WB analysis, a fraction of cytoplasmic and nuclear samples were collected in RIPA buffer (50 mM Tris, pH.7.5, 150 mM NaCl, 0.5% NP-40, 0.5% NaDoc, 1% Triton, Protease inhibitors in MilliQ). Nuclear sample was lysed by passing through a 27 g needle (BD Plastipak) for five times followed by sonication. 12 µl of the cytosol and 6 µl of nuclear protein samples were resuspended in 4 × NuPAGE LDS Sample buffer (Catalogue# NP0007, ThermoFisher Scientific) and heated at 95 °C for 5 min. The samples were separated in SDS-PAGE gels (8%) for approximately 1 h at 160 V, until the dye ran out of the gel. Separated proteins were transferred onto nitrocellulose blotting membrane (GE Healthcare) for 1 h at 100 V under cold conditions. Then, blots were blocked for 1 h at room temperature (RT) in 5% milk powder in 1 × TBS-Tween. Blots were incubated overnight at 4 °C with antibodies against the nuclear-enriched protein fibrillarin (FBRL) (1:1000, C13C3, Catalogue# 2639S, Cell Signaling Technology) or the cytoplasmic-enriched protein *β*-tubulin (1:2000, Catalogue# T4026, Sigma-Aldrich) in blocking buffer. ﻿Blots were stained with peroxidase-conjugated secondary antibodies (1:30000 for HRP-anti-mouse, Catalogue#1705046, Bio-Rad; 1:50000 for HRP-anti-rabbit, Catalogue#1705047, Bio-Rad) for 1 h at RT in 1 × TBS-Tween, and signal was detected by incubating blots with Pierce ECL substrate (Catalogue# 34076, Thermo Fisher Scientific). Images were acquired using a FluorChem M imaging system (Protein Simple). Different antibodies and conditions used for WB are in Supplementary excel file (Tab3).

RNA levels of nuclear-enriched markers (*NEAT1*, *GOMAFU*, and *pre-GAPDH*) and cytoplasmic-enriched markers designed to amplify an intron spanning amplicon (*GAPDH*) were measured by RT-qPCR, both in cytoplasmic and nuclear preparations. Briefly, 100 ng of total RNA reaction was reverse transcribed using the SuperScript III First-Strand Synthesis System (Catalogue# 18080051, ThermoFisher Scientific) with random hexamers, according to the manufacturer’s instructions. RT-qPCR quantification of mRNA was conducted using FastStart Universal SYBR Green Master (Rox) (Roche) for all samples, in triplicates, in a QuantStudio™ 6 Flex Real-Time PCR System (ThermoFisher Scientific). Real-time reactions were carried out as follows: pre-denaturation at 95 °C for 10 min, followed by 40 cycles at 95 °C for 15 s and 60 °C for 1 min. Melting curves were generated for the final PCR products by decreasing the temperature to 60 °C for 1 min followed by an increase in temperature to 95 °C. Primer sequences used for RT-qPCR reactions are listed in Supplementary Table 1.

### Library preparation and small RNA sequencing

For miRNA library construction and sequencing, small RNAs < 200 nucleotides were purified using the *mir*Vana microRNA isolation kit (Catalogue# AM1560, Ambion, ThermoFisher Scientific). One hundred nanograms of small RNA was prepared for sequencing using the TruSeq Small RNA preparation kit (Illumina, San Diego, California, USA). Small RNA sequencing was performed on an Illumina HiSeq 4000 sequencer at the Beijing Genomics Institute (BGI), Hongkong, China.

### RNA sequencing analysis

#### miRNA analysis

Following trimming of low-quality bases and adapter sequences with FASTQ-MCF (version 0.0.13), processed reads were mapped using Bowtie. MiRNA alignment was performed against the human reference from miRBase (release v21). For differential expression analysis, count data for miRNAs were analyzed for differential expression (DE) in R using the Bioconductor package EdgeR version 3.12.1 [103] with the trimmed mean of *M*-values (TMM) normalization method [104]. Adjusted *P *values for multiple testing were calculated using the Benjamini–Hochberg false discovery rate (FDR) and only genes with an FDR < 0.05 were considered significantly differentially expressed. All raw files, count data, and the DE data are deposited at Gene expression Omnibus (GEO) with accession number GSE245228. Results from the data analysis and the list of DE genes are provided in Supplementary excel file (Tab4 to Tab11).

#### IsomiR analysis

IsomiR analysis was performed using the IsomiRage Java tool [86]. Briefly, -map bowtie output files were used as inputs for the Isomirage pipeline that returned a table containing the number of reads for each isoform in each biological sample. Significant changes in isoform expression were detected using EdgeR, as described above. Summarizing heatmaps and plots were generated in R using pheatmap and ggplot2, respectively. All raw files, count data and the DE data are deposited at Gene expression Omnibus (GEO) with accession number GSE245228. List of DE isomiRs are provided in Supplementary excel file (Tab12 to Tab17).

#### SnoRNA analysis

**Fig. 1:**
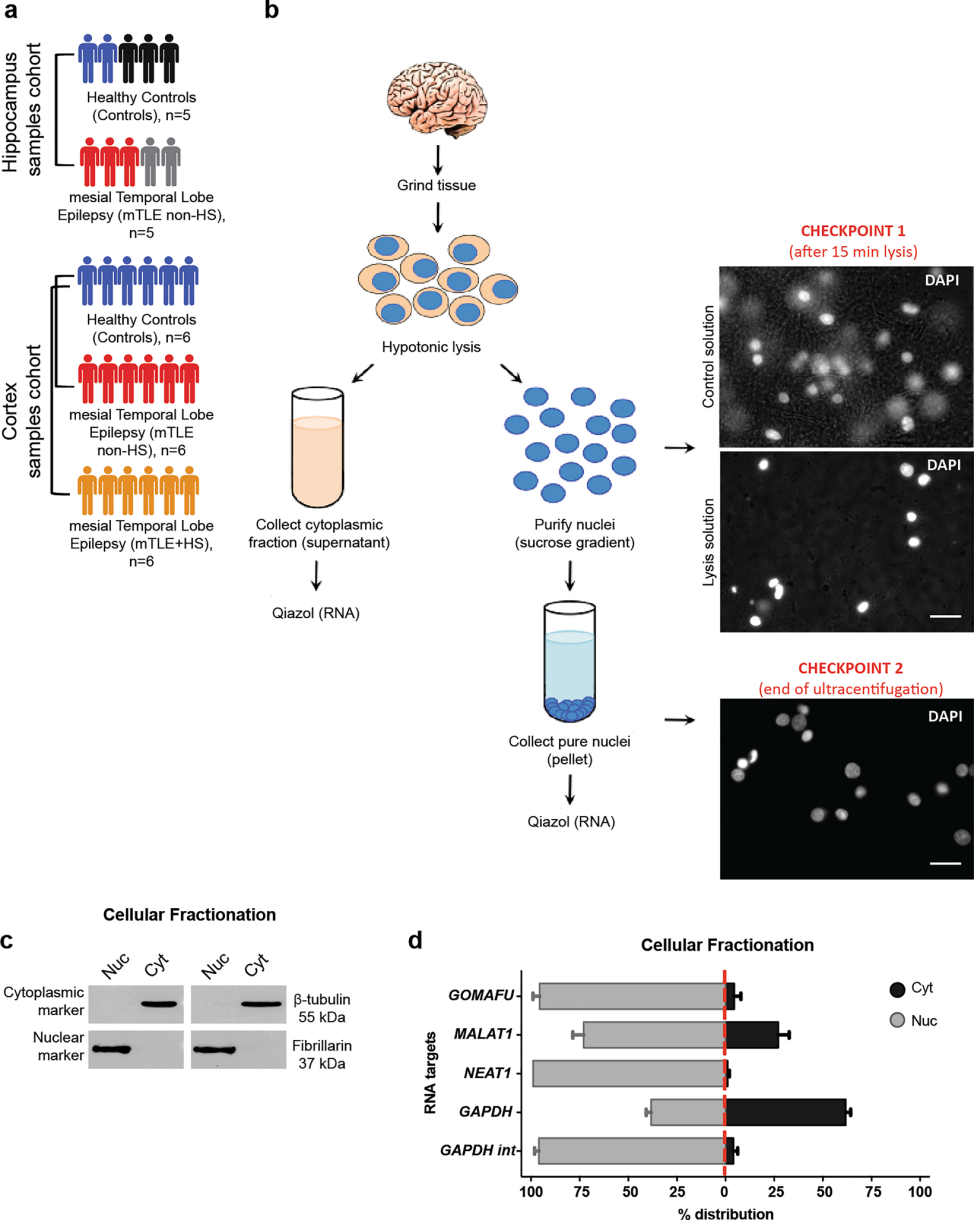
Nuclear fractionation of human brain tissue. **a** Schematic illustrating the different cohorts (resected brain material from mTLE patients and postmortem control tissue) used for small RNA-seq of hippocampal and cortical tissue. *non-HS*, no hippocampal sclerosis; + *HS*, hippocampal sclerosis. **b** Overview of the key steps of the cell fractionation procedure to isolate pure nuclear and cytoplasmic RNA samples. **c** Western blot analysis of nuclear (Nuc) and cytoplasmic (Cyto) fractions. Fibrillarin and *β*-Tubulin were used as nuclear and cytoplasmic markers, respectively. Consistent with its sub-cellular localization, fibrillarin was specifically detected in the nuclear fraction, whereas anti-*β*-tubulin antibody showed signal only in cytoplasmic fractions. *n* = 3 experiments were performed with similar results with both hippocampal and cortical tissue (*n* = 1 each) samples. **d** Quantitative RT-PCR for *GOMAFU*, *MALAT1*, *NEAT1*, *GAPDH* and *GAPDHint* (an intron retained variant of *GAPDH*) on nuclear and cytoplasmic RNA samples. Higher levels of *GAPDH* were reported in the cytoplasmic fraction, whereas the other genes were enriched in nuclear fractions. Enrichment is represented as a percentage of the distribution (% distribution) of a marker gene across the nuclear and cytoplasmic compartments. *n* = 3 experiments were performed with similar results, with *n* = 2 mTLE non-HS and one control human tissue samples. Data shown as means + SD

DE analysis for all ncRNAs with a focus on snoRNAs was performed by analyzing the small RNA-seq datasets generated from nuclear and cytoplasmic compartments of hippocampal control and mTLE non-HS samples (Fig. 1). The quality of obtained fastq files was assessed by fastQC version 0.10.0 [131]. Sequenced reads were trimmed (Illumina RA3 adapter: TGGAATTCTCGGGTGCCAAGG), filtered (> 18nt) using Cutadapt 3.4 [76] and assigned using Salmon 1.5.2 to a built index for human ncRNA (Biomart Ensembl human version 99) [96]. Before mapping, the k-mer size was optimized using randomly selected samples from cytoplasm and nucleus and different k-mer sizes to build indexes (from 13 to 23, odd numbers). The mapping rate and the average of transcripts per million counts (TPM) were compared to select the best experimental design (Supplementary Table 2 and 3). Furthermore, we also assigned the same samples to a snoRNA index obtained from RNA central database using different k-mer sizes [97]. After optimization and mapping using k-mer 13 as default, read counts were combined accordingly to snoRNA classes (Supplementary excel file (Tab19)). DE genes were detected using the raw counts from Salmon through DESeq2 package in R studio (version 1.4.1103) following the default parameters, including Benjamin–Hochberg adjusted *p* value for false discovery rate (FDR). Genes were only considered DE if adjusted *p* value < 0.05 and log2 fold change > 1. Four HC controls and five HC mTLE non-HS small RNA-seq datasets were included in the analysis. One of the control samples displayed variation in clustering on the PCA and heat maps and was hence excluded from the final analysis (data not shown). Results from the data analysis and the list of DE genes are provided in Supplementary excel file 4 (Tab19 and Tab20).

### Prediction of 2′-O-ribose methylation (Nm) sites

To predict the methylation function of DE snoRNAs that are also interacting with *miR-92b*, we predicted methylation sites for *snoRD14E*, *snoRD35B*, and *snoRD38A* using the Plexy tool [59]. SnoRNA sequences were given as input, with annotated header informing the location of D- and/or D prime-box. Further methylation sites were predicted by specifying the target database; cDNA sequences (for mRNA targets) and ncRNA (for rRNA and other RNA classes) (Biomart Ensembl human version 99). The snoRNA sequences used for prediction, as well as the obtained list of predicted methylation sites are provided in Supplementary excel file (Tab21 to 24).

### Validation of DE miRNAs by quantitative RT-PCR

For validation and replication of sequencing data, we performed RT-qPCR for 8 selected miRNAs (5 significant DE nuclear-enriched miRNAs with highest mean expression and 3 significant DE cytoplasmic-enriched miRNAs with highest mean expression). Ten nanograms of total RNA reaction was reverse transcribed using the miRCURY LNA RT Kit (Catalogue# 339340, Qiagen) according to the manufacturer's instructions. Real-time RT-PCR (RT-qPCR) for quantification of the miRNAs amount was conducted using miRCURY LNASYBR Green PCR Kit (Catalogue# 339345, Qiagen) for all samples, in triplicate, with a QuantStudio™ 6 Flex Real-Time PCR System (ThermoFisher Scientific). miRCURY LNA miRNA PCR assay primers (Catalogue# 339306, Qiagen) were used to detect the expression levels of miRNAs. Real-time reaction was carried out as follows: pre-denaturing at 95 °C for 10 min, followed by 40 cycles at 95 °C for 10 s and 56 °C for 1 min. Melting curves were generated for the final PCR products by decreasing the temperature to 60 °C for 1 min followed by an increase in temperature to 95 °C. Real-time PCR MyIQ software (Bio-Rad, RRID:SCR_019736) was used to determine the amplification cycle in which product accumulation was above the threshold cycle values (CT). Relative quantification was determined using the 2^−ΔCT^ method [72] and normalized to the endogenous 5S. Unpaired Welch t test was used for multiple comparisons with a 95% confidence cutoff. We considered the difference between comparisons to be significant when *P* < 0.05 (*P < 0.05, **P < 0.01, ***P < 0.005, ****P < 0.001).

### miRNA in situ hybridization and co-labeling with antibodies

Non-radioactive in situ hybridization (ISH) was performed as described previously [89, 124]. Tissue sections of fresh-frozen specimens from at least three or four patients and postmortem controls were used for ISH. Similarly, ISH was performed on paraffin tissue sections from at least three pilocarpine rats and their corresponding controls for each timepoint. For fresh-frozen tissue sections, 16 µm thick sections from human tissue were collected on adhesive-coated glass slides and stored at – 80 °C until used. For paraffin embedded tissue sections, 7 µm-thick sections were deparaffinized and rehydrated before continuing with ISH.

Tissue sections were fixed by treatment with 4% PFA for 10 min at RT, acetylated (10 min at RT), and treated with proteinase K (5 µg/ml in 1 × PBS) for 5 min at RT. Following three washes in 1 × PBS prehybridization was performed for 1 h at RT. Hybridization of the probe was performed by incubating with 10 or 20 nM of double-digoxigenin (DIG) (3’ and 5’) labeled locked nucleic acid (LNA)-modified probe for *hsa-miR-92b-3p* (20 nM), *hsa-miR-124-3p* (10 nM), *hsa-miR-423-3p* (20 nM), or LNA-DIG scrambled miR-probe (Catalogue# 339111, Qiagen) overnight at 55 °C. Following, slides were washed in 0.2 × SSC at 60 °C for 1 h and blocked in B1 solution (0.1 M Tris pH 7.5, 0.15 M NaCl) for 1 h at RT. Finally, sections were incubated with anti-digoxigenin-AP Fab-fragments antibody (1:2500, Catalogue# 11093274910, Roche) and 10% FCS in B1 buffer overnight at 4 °C. Slides were washed with B1 and treated with NBT and BCIP substrate (NBT/BCIP stock solution, Catalogue# 11681451001, Roche) in B3 buffer (0.1 M Tris pH 9.5, 0.1 M NaCl, 50 mM MgCl_2_) from 5 to 20 h at RT. Staining was monitored regularly and stopped by washing in 1 x PBS. Sections were mounted using Fluorsave™ reagent (Catalogue# 345789, Merck Millipore) or antibody staining was performed. Following ISH tissue sections were incubated with FBRL primary antibody (1:400, Cell Signaling Technology) diluted in 0.3% Triton-X-100, 0.2% bovine serum albumin (BSA), 3% normal goat serum (NGS) in 1 × PBS overnight at 4 °C. Sections were washed in 1 × PBS and incubated with secondary antibody (1:500, AlexaFluor-488, Catalogue# ab150077, Abcam) in 1 × PBS for 1.5 h at RT. Finally, nuclei were stained with 4',6-diamidino-2-phenyindole (DAPI, 1x, Catalogue# D9564, Sigma-Aldrich) in 1 × PBS for 10 min at RT and sections were mounted using Fluorsave™ reagent (Merck Millipore) mounting medium. Images were acquired with AxioScope microscope (Zeiss) and processed using ImageJ/Fiji ([109]RRID:SCR_002285).

### Cell culture and cell lines

Mouse Neuro2A neuroblastoma cells (RRID:CVCL_0470) were purchased from ATCC (CCL-131™). Cells were cultured in Dulbecco's modified Eagle's medium (DMEM), containing 10% FBS (ThermoFisher Scientific) and penicillin/streptomycin (100 U/ml and 100 mg/ml, respectively) at 37 °C and 5% CO2. Transient expression of biotinylated mimics was conducted for 6 h using the TransIT-X2 Dynamic Delivery System (Catalogue# MIR6004, Mirus) according to the manufacturer’s instructions. After 6 h, cells were washed with 1 × PBS and returned to regular medium for 2 days. For immunoprecipitation, 25 cm plates at ∼ 80–90% confluence were transfected with biotinylated mimics (25 nM final concentration) and 120 µl of TransIT-X2 Dynamic Delivery System, to reach approximately ∼ 85–90% transfection efficiency.

### Nuclear-cytoplasmic fractionation Neuro2A cells and RNA co-immunoprecipitation

Fractionation of Neuro2A cells was performed using the protocol described for human brain tissue with some modifications. Briefly, Neuro2A cells ready to be fractionated were trypsinized, washed twice with pre-chilled PBS, and lysed in Hypotonic Lysis Buffer (HLB) [10 mM Tris (pH 7.4), 3 mM CaCl_2_, 2 mM MgCl_2_, 1% Nonidet P-40, Protease inhibitor, 60 U SUPERase-In/ml] for 10 min on ice to lyse. Approximately 10–30 strokes of a tight fitted glass Douncer were applied to enhance the lysis process. Following gentle centrifugation (500 *g* × 5 min at 4 °C), the semi-pure nuclei (pellet) were resuspended in 2 ml Sucrose Buffer I [0.32 M Sucrose, 5 mM CaCl_2_, 3 mM Mg(CH_3_COO)_2_, 0.1 mM EDTA, 10 mM Tris (pH 8.0), 0.5% Nonidet P-40, 1 mM DTT, 25 U SUPERase-In/mL] and layered on top of a 2.0 M Sucrose Buffer II [2.0 M Sucrose, 3 mM Mg(CH_3_COO)_2_, 0.1 mM EDTA, 10 mM Tris (pH 8.0), 1 mM DTT, Protease Inhibitor, 35 U SUPERase-In/ml], and 2.3 M Sucrose Buffer III [2.3 M Sucrose, 3 mM Mg (CH_3_COO)_2_, 0,1 mM EDTA, 10 mM Tris (pH 8.0), 1 mM DTT, 35 U SUPERase-In/ml, Protease Inhibitor], respectively, in ultracentrifugation tubes (Beckman Coulter). Samples were centrifuged using a Ti70 rotor at 30,000 *g* at 4 °C for 45 min. Pure nuclei were collected and stored in Qiazol (Qiagen), for RNA analysis, or in RIPA buffer (50 mM Tris, pH.7.5, 150 mM NaCl, 0.5% NP-40, 0.5% NaDoc, 1% Triton, Protease inhibitor (Roche) in MilliQ) for co-immunoprecipitation of biotinylated mimics. RNA co-immunoprecipitation was performed as described previously [124]. The efficiency of the fractionation protocol was checked by protein and RNA assays. Western blotting was performed probing both nuclear and cytoplasmic protein samples against the nucleus-enriched protein fibrillarin and the cytoplasm-enriched protein *β*-tubulin. RNA levels of nuclear-enriched markers (*Neat1, Gomafu, pre-Gapdh*) and cytoplasmic-enriched markers (*Gapdh*) were measured by RT-qPCR in both cytoplasmic and nuclear preparations. Briefly, 100 ng of total RNA reaction was reverse transcribed using the SuperScript III First-Strand Synthesis System (ThermoFisher Scientific) with random hexamers according to the manufacturer's instructions. RT-qPCR for quantification of mRNA was conducted using FastStart Universal SYBR Green Master (Rox) (Roche) for all samples, in triplicate, with a QuantStudio™ 6 Flex Real-Time PCR System (ThermoFisher Scientific). Real-time reaction was carried out as follows: pre-denaturing at 95 °C for 10 min, followed by 40 cycles at 95 °C for 15 s and 60 °C for 1 min. Melting curves were generated for the final PCR products by decreasing the temperature to 60 °C for 1 min followed by an increase in temperature to 95 °C. The primer sequences used for RT–qPCR reactions are shown in Supplementary Table 1.

### RNA sequencing and analysis of immunoprecipitated samples

Three samples per group were used for RNA sequencing. The NEBNext Ultra Directional RNA Library Prep Kit for Illumina (Catalogue# NEBE7760, New England Biolabs) was used to process the samples. Sample preparation was performed according to NEBNext Ultra Directional Library Prep Kit for Illumina (Catalogue# NEBE7420S/L, New England Biolabs) protocol. Briefly, using oligo-dT magnetic beads, mRNA was isolated from total RNA, followed by mRNA fragmentation and cDNA synthesis. The resulting cDNA was ligated with the sequencing adapters followed by PCR amplification. The yield and quality of the samples was estimated using a Fragment analyzer, where the size of the resulting products was consistent with the size distribution showing a broad peak between 300 and 500 bp. Subsequently, clustering and DNA sequencing were performed using the Illumina cBot and HiSeq 4000, according to the manufacturer’s protocol. HiSeq control software HCS v3.3.52 was used. The sequencing experiments were performed at GenomeScan B.V., Leiden, The Netherlands. Image analysis, base calling, and quality check were performed with the Illumina data analysis pipeline RTA v2.7.3 and Bcl2fastq v2.17. Quality filtered sequencing tags were stored as raw data for further analysis. Adaptor reads were trimmed using Trimmomatic v0.30. Reads were mapped to mouse GRCm38 (mm10) genome using Tophat2. From the mapped reads, transcripts were assembled using Cufflinks. Differential gene expression analysis was performed using Ciffdiff (Cufflinks package). Using R package cummeRbund output plots were made. All raw files, count data and the DE data are deposited at Gene expression Omnibus (GEO) with accession number GSE269625. Panther overrepresentation analysis was performed for Reactome pathways on the significantly DE genes, and FISHER test and FDR correction were applied to obtain significantly enriched pathways [79, 119]. Results from the data analysis and the list of DE genes are provided in Supplementary excel file (Tab25 and Tab26).

### Immunohistochemistry and western blot analysis

Immunohistochemistry and western blotting on human tissue were performed as previously described [124]. For immunostaining, 16 µm-thick human tissue sections were blocked for 1 h at RT and incubated with primary antibodies: nucleophosmin (NPM1) (FC-61991, Catalogue# 32–5200, ThermoFisher Scientific), FBRL (Cell Signaling Technology), nucleolin (C23) (MS-3 (Catalogue# sc-8031, Santa Cruz Biotechnology), or CBX4 (E6L7X, Catalogue# 30,559; Cell Signaling Technology) antibodies diluted in 0.2% BSA, 3% NGS in 1 × PBS overnight at 4 °C. Tissue samples were washed and incubated for 1.5 h at RT with the appropriate secondary antibodies (Alexa Fluor 488 or Alexa Fluor 568, (Catalogue#s ab150077, ab175471, ab175473, Abcam or Catalogue# A-11029, ThermoFisher Scientific) diluted in 1 × PBS. Finally, slides were washed in 1 × PBS and stained for nuclei with DAPI for 10 min at RT and mounted with ProLong™ Gold mounting medium (Catalogue # P0144, ThermoFisher Scientific). Images were acquired with confocal microscope with either 40 × or 63 × oil immersion objective (LSM880, Carl Zeiss) and processed using Fiji.

For analysis of total protein levels in human samples and Neuro2A cells transfected with guide RNAs targeting *Npm1*, protein concentration was estimated with the BCA assay. Unless specified otherwise, 10 µg of protein lysate was diluted with 4 × Nu-PAGE sample buffer (with 10% *β*-mercaptoethanol in MilliQ) and boiled for 5 min at 95 °C. SDS-PAGE gels were used to resolve the proteins, after resolving proteins were transferred onto nitrocellulose membrane (GE healthcare). Blots were blocked with 5% milk powder in 1 × TBS-Tween for 1 h at RT, following which incubated with respective primary antibody in blocking solution ON at 4 °C. Primary antibodies used for WB, FBRL, *β*-actin (Catalogue# A5316, Sigma-Aldrich), C23, NPM1, CBX4, and CBX8 (D208C; Catalogue# 14696; Cell Signaling Technology). Details of antibodies, dilutions, and incubation conditions for immunostaining and western blotting are provided in Supplementary excel file (Tab3).

Following washes, blots were incubated with peroxidase-conjugated secondary antibodies [HRP-anti-rabbit (Bio-Rad)] or HRP-anti-mouse (Bio-Rad) in 1 × TBS-Tween for 1 h at RT, followed by incubating with Pierce ECL substrate (ThermoFisher Scientific) to detect signal and images were acquired by FluorChem M imaging system (Protein Simple). Individual band intensities were measured using ImageJ from the protein of interest and corresponding loading control *β*-actin band. Differences in expression were estimated after normalizing to *β*-actin and statistical significance was estimated by Mann–Whitney test using Graphpad Prism (version 9) software (RRID:SCR_002798). For assessing FBRL protein levels, blots were blocked in Supermix blocking solution (Tris 50 mM, NaCl 154 mM, 0.25% gelatin, 0.5% Triton X-100 in MilliQ, pH 7.4) after transfer for 10 min at RT and incubated with rabbit FBRL and mouse *β*-actin antibody ON at 4 °C. The next day, blots were washed in 1 × TBS-tween and incubated with secondary antibodies coupled with IR dyes (antirabbit-IRdye 800 1:5000 (Catalogue# 926–32211) and anti-mouse-IRdye 700 1:2000 (Catalogue# 926–68070); LI-COR Biosciences) in 1 × TBS-Tween for 1 h at RT. Finally, signals were obtained by scanning on an Odyssey Clx imaging system (Li-COR Biosciences) using Li-COR Image studio version 3.1 software (RRID: SCR_015795). Band intensities were measured and the relative expression between conditions was estimated after normalizing to *β*-actin. Statistical significance was calculated with the Mann–Whitney test using Graphpad Prism (version 9) software.

### Image analysis

Unless specified otherwise, image quantification was performed in the different Cornu Ammonis (CA) regions of HC and only cells that resembled pyramidal neurons were included in the analysis. Details of patient and control samples used for analysis are provided in Supplementary excel file 1 (Tab1 and Tab2). For estimating the number of nucleoli with miRNA signal in human TLE and AD tissue, cells with *miR-92b* or *miR-124* ISH signals were counted in the CA1, CA3, and CA4 hilar regions of the HC. The average number of cells per region was estimated and statistical significance was calculated. Tissue sections from four controls, four mTLE non-HS, four mTLE + HS, three AD (Braak6), and two AD (Braak5) patients were used for the analysis.

For measuring the size and shape of nucleoli based on NPM1 immunostaining in control, mTLE non-HS and mTLE + HS tissue, confocal images acquired using 40 × objective were processed using ImageJ. Area of NPM1 staining marking neuronal nucleoli was measured and plotted for each condition. For the shape of the nucleoli, the circularity plugin on ImageJ was used, and values closer to 1 indicate circular shape, whereas lower value indicate more elongated or distorted shapes. Tissue sections from four controls, four mTLE non-HS, and four mTLE + HS were used for the analysis.

For estimating the size of nucleoli in rat pilocarpine HC tissue, confocal z-stack images were acquired using a 40 × objective with similar settings for the different conditions. ImageJ was used for processing and thresholding the images to obtain a clear NPM1-positive area as circles that label the GC area of the nucleoli. Next, using the Particle analysis plugin, particles > 1 µm were measured for estimating the average size of NPM1-positive area per image with clearly distinct nucleolus-like structures. Finally, the average nucleolus area between control and SE + conditions was estimated at different timepoints in different regions of the HC. For 2 wk, four controls and four SE + animals, and for 19 wk two controls and three SE + animals with images from both right and left HC were used for analysis. For quantification of nuclear *miR-92b* expression levels in control and pilocarpine SE + animals, mean ratios of miRNA ISH signal were estimated by densitometry analysis measuring *miR-92b* signal in the nucleus based on DAPI-positive area in the blue channel in the same cell using a previously described formula [128]. Ten images from three control animals at 2 weeks and ten images from each of three SE + animals at 2 weeks, 4 weeks, 8 weeks, and 19 weeks after SE were used for the analysis.

For quantification of the localization, intensity, and size of nucleoli based on C23 fluorescence, confocal images acquired using a 63 × objective were processed with ImageJ in each sub-compartment (Nucleus, Nucleoli, and Soma). The area of each sub-compartments was drawn manually, based on C23 signal in the whole cell, DAPI for nucleus and C23 signal for nucleoli, as shown in Fig. 6C. From these regions of the cell, area, mean fluorescence, and integrated density were estimated. Then, using the corrected total cell fluorescence (CTCF) formula [CTCF = Integrated Density – (Area of selected cell x Mean fluorescence of background readings)], overall C23 intensity differences were calculated in the different sub-regions of the HC between controls and patient samples. From these measurements, the mean intensity of C23 signal for individual compartments was estimated in the different sub-regions as normalized mean intensity ratio. Finally, the area of the nucleus and nucleolus in the different sub-regions were estimated between conditions based on the area measurements. HC tissue from four controls and three mTLE non-HS patients were used for the analysis.

For CBX4 co-localization analysis, confocal *z*-stack images acquired with similar settings among different groups using 63 × objective were used. Images were processed using the Just Another Co-localization (JACoP v2.0) plugin on ImageJ/Fiji [11]. Manual threshold was applied and the Mander’s co-localization coefficient was estimated by calculating the fraction of A overlapping over B between the different groups. Two Cx tissue samples adjacent to a tumor, three mTLE non-HS Cx, and three HC tissue were used for the analysis. Staining with CBX4 antibody on control tissue resulted in unspecific staining, which could be due to longer fixation times of the controls. Similarly, CBX8 antibody resulted in unspecific staining in either tissue types. Hence, for quantification of protein mis-localization, control and control and mTLE non-HS tissue were not included in the CBX4 and CBX8 analysis, respectively.

### Statistical analysis

Statistical analysis was performed using GraphPad Prism (version 9), and *P* < 0.05 was considered significant. Wilcoxon Mann–Whitney test or unpaired t test was used for comparing two groups. ANOVA was used for comparing more than two groups. *P* values and the number of samples ‘*n*’ used for each experiment are mentioned in the figure legend. Error bars represented with the graphs are either standard deviation (SD) or standard error of the mean (SEM).

## Results

### Small RNA sequencing of sub-cellular fractions in mTLE

Our previous work indicated that mTLE is characterized by changes in sub-cellular mRNA expression in the brain [122] and identified changes in the sub-cellular localization of specific miRNAs [56]. However, the full extent of changes in miRNA localization remained unclear. Therefore, to gain a more comprehensive understanding of the sub-cellular distribution of miRNAs in human mTLE patient brain tissue, small RNA sequencing (RNA-seq) was performed on: (1) five mTLE non-HS and five control hippocampus (HC) samples, and (2) six mTLE non-HS, six mTLE + HS, and six control cortex (Cx) samples (Fig. 1a). Nuclear and cytoplasmic fractions of human brain tissue from patients and controls were successfully isolated by tissue lysis in hypotonic lysis buffer followed by ultracentrifugation (Fig. 1b). Fractionation success was examined at both the protein and RNA level. The cytoplasmic protein *β*-tubulin was only observed in cytoplasmic fractions, whereas the nucleus-enriched protein fibrillarin (FBRL) was observed in nuclear fractions (Fig. 1c). At the RNA level, the cytoplasmic marker gene *GAPDH* was enriched in cytoplasmic fractions, whereas in nuclear fractions, several nucleus-specific lncRNAs were strongly enriched (*GOMAFU*, *MALAT1*, *NEAT1)* in addition to *GAPDH* intronic transcripts (Fig. 1d).

Small RNA-seq on RNA purified from sub-cellular fractions resulted in on average 21.1 million high-quality reads in the cytoplasm and 15.4 million reads in the nucleus for Cx samples. For HC samples, 16.7 and 15.6 million reads were obtained from cytoplasmic and nuclear fractions, respectively [Supplementary Fig. 1a, 1d; Supplementary excel file (Tab4)]. No significant differences in the total number of miRNA counts and the number of miRNAs detected per sample were observed between the different cytoplasmic or nuclear Cx or HC samples [Supplementary Fig. 1b, 1c, 1e, 1f; Supplementary excel file (Tab4)]. Overall, these data support our ability to generate sub-cellular fractions from freshly resected and postmortem brain tissue and show that miRNA counts of the sub-cellular fractions isolated were similar between different control and patient groups.

Postmortem delay is a common feature of control tissue samples obtained from brain banks [36]. It is often not clear to which extent postmortem delay in control tissues affects transcript levels and differential gene expression analysis when compared with samples collected without postmortem delay. To estimate this potential confounding effect for our study, freshly resected Cx tissue was subjected to postmortem delay treatment. Briefly, tissue was cut into two halves, where one half (pre-) was stored immediately on dry ice and then at − 80 °C until processing. The second half (post-) was incubated at 4 °C in saline for 24 h to mimic postmortem conditions observed for tissue samples collected from brain banks [33, 110]. Our previous work did not detect significant differences in overall transcriptomic profiles between pre- and postmortem samples for nuclear and cytoplasmic fractions [122]. In line with this observation, small RNA-seq of pre- and postmortem samples did not reveal strong differences in the overall coverage and mapping of reads. On average, 17.6 and 15.5 million reads were detected in pre- and postmortem cytoplasmic fractions, respectively, and 18.5 and 16.2 million reads in pre- and postmortem nuclear fractions, respectively [Supplementary Fig. 1a, 1d; Supplementary excel file (Tab4)]. No significant changes in miRNA expression were observed in the same sample before and after experimental postmortem delay, neither in cytoplasmic nor in nuclear fractions (Supplementary Fig. 1g–h). In comparison, significant differences in miRNA gene expression were observed between control and mTLE non-HS HC samples (Supplementary Fig. 1i–j). Together, our data do not support large differences in miRNA profiles as a result of postmortem delay.

**Fig. 2:**
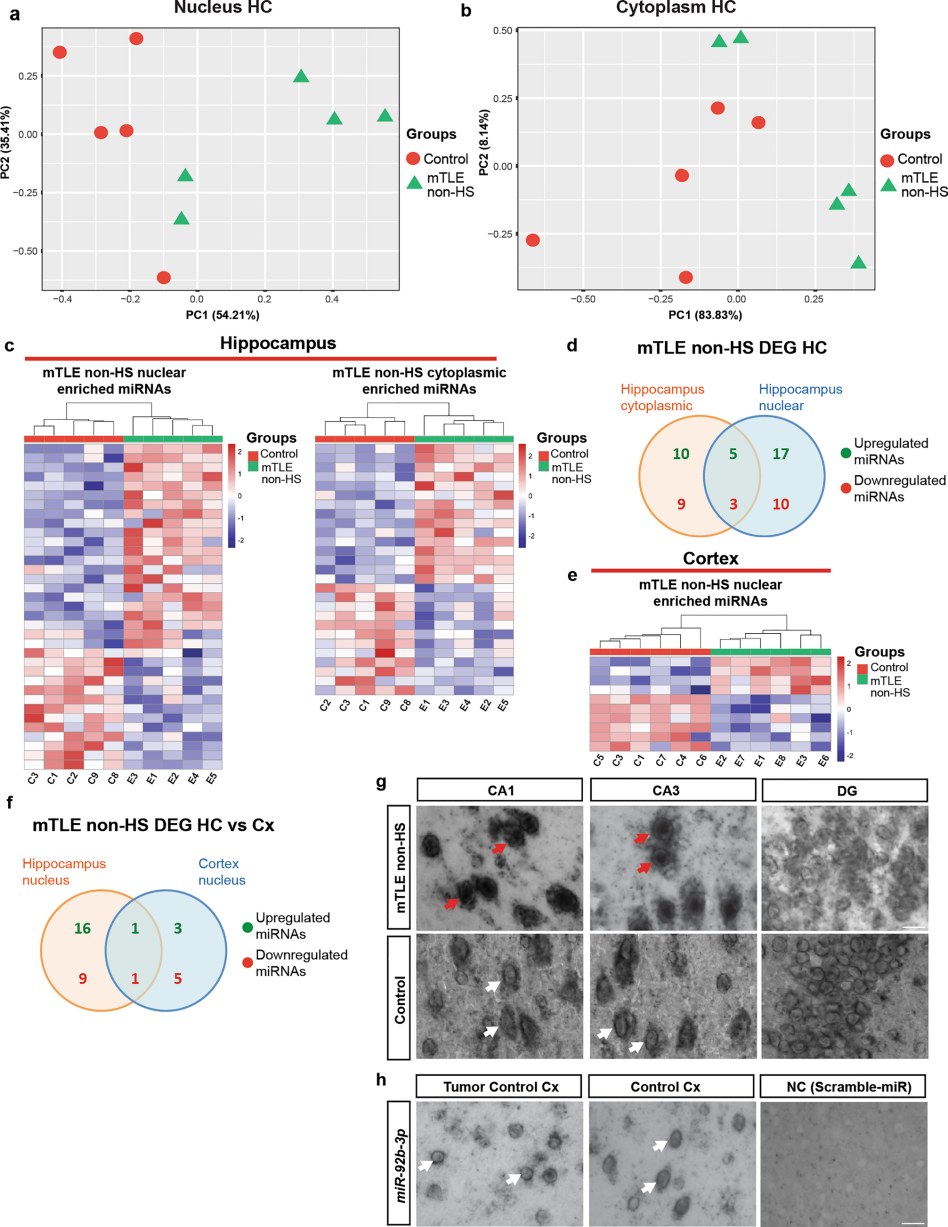
Compartment-specific changes in miRNA expression in human mTLE brain tissue. **a, b** Principal Component Analysis (PCA) for hippocampal (HC) nucleus and cytoplasmic samples from controls and mTLE non-HS patients (*n* = 5 each) based on deregulated miRNA expression detected by small RNA-seq. **c** Heatmaps showing differentially expressed miRNAs in nuclear and cytoplasmic samples from the HC of 5 controls (C1, C2, C3, C8, C9) and 5 mTLE non-HS patients (E1–5) determined by small RNA-seq. Hierarchical clustering is shown on top. For DE miRNAs see Supplementary excel file (Tab5 and Tab6). **d** Venn diagram showing the overlap of differentially expressed miRNAs in the HC cytoplasmic and nuclear fractions of mTLE non-HS patients. **e** Heatmap showing differentially expressed miRNAs in nuclear fractions of the cortex (Cx) of 6 controls (C1, C3–7) and 6 mTLE non-HS patients (E1–3, E6–8). For DE miRNAs, see Supplementary excel file (Tab7). **f** Venn diagram showing the overlap of differentially expressed miRNAs in nuclear fractions from mTLE non-HS HC and Cx tissue. **g** Representative images showing in situ hybridization for *miR-92b-3p* on coronal sections of postmortem control and mTLE non-HS HC [CA1, CA3, and dentate gyrus (DG) regions]. Red arrows indicate nuclear accumulation of *miR-92b-3p* in CA neurons. White arrows indicate normal cytoplasmic localization. Scale bar, 50 μm. *n* = 2 ISH experiments were performed with similar results. *n* = 3 control, *n* = 3 mTLE non-HS. **h** Representative images showing in situ hybridization (ISH) for *miR-92b-3p* on coronal sections on unaffected Cx tissue removed during tumor surgery (tumor control) or postmortem control Cx (control). NC, scrambled control probe. White arrows indicate normal *miR-92b-3p* cytoplasmic expression. Scrambled control probe did not yield specific signals. Scale bar, 50 μm. *n* = 1 ISH experiment, with *n* = 2 tumor control Cx, *n* = 3 control Cx

Further analysis of the RNA-seq data revealed separate clustering of control and mTLE non-HS HC samples for both nuclear and cytoplasmic fractions (Fig. 2a, b) and a higher number of deregulated miRNAs in mTLE HC nuclear as compared to cytoplasmic fractions (Fig. 2c). Overall, 22 miRNAs were upregulated and 13 miRNAs downregulated in the nucleus of mTLE HC samples, while 15 miRNAs were upregulated and 12 downregulated in the cytoplasm. Comparison of deregulated miRNAs in HC samples identified five commonly upregulated (i.e., in cytoplasm and nucleus; *hsa-miR-4454*, *hsa-miR-1260b*, *hsa-miR-486-3p*, *hsa-miR-27a-5p, and hsa-miR-let-7c-3p*) and three downregulated miRNAs (*hsa-miR-3615*, *hsa-miR-1298-5p*, *hsa-miR-1911-5p*) in mTLE non-HS HC cytoplasmic and nuclear compartment (Fig. 2d). Changes in miRNA in Cx samples were less pronounced where sample clustering was not well segregated and only a few miRNAs showed altered expression in nuclear, but not cytoplasmic, samples of mTLE non-HS patients as compared to control (Fig. 2e; Supplementary Fig. 2a–d). In mTLE + HS Cx samples, sample clustering and segregation between groups were also not very well defined, but a few miRNAs were deregulated in both nuclear and cytoplasmic compartments (Supplementary Fig. 2e–h). mTLE non-HS HC and Cx nuclear samples shared one upregulated (*hsa-miR-4485-3p*) and one downregulated (*hsa-miR-320c*) miRNA (Fig. 2f).

In addition to canonical miRNAs, we analyzed the changes in abundance of isomiRs in mTLE samples. IsomiRs are miRNA isoforms that differ in length and sequence because of posttranscriptional non-templated nucleotide additions or deletions on either side of the canonical miRNA sequences [14, 85, 132]. In line with the miRNA data, a large number of deregulated isomiRs was detected in the nuclear fraction of mTLE non-HS HC samples, while fewer isomiRs showed altered expression in cytoplasmic samples [Supplementary Fig. 3a; Supplementary excel file (Tab12 to Tab17)]. Only a few differentially expressed isomiRs were detected in Cx nucleus fractions of mTLE non-HS samples (Supplementary Fig. 3b). Comparison of HC cytoplasmic and nuclear samples revealed two commonly upregulated isomiRs and ten isomiRs that were downregulated [Supplementary Fig. 3c; Supplementary excel file (Tab12 to Tab17)]. Further, the nuclear fractions of HC and Cx samples shared two downregulated isomiRs but no upregulated isomiRs [Supplementary Fig. 3d; Supplementary excel file (Tab12 to Tab17)].

Finally, RT-qPCR was used to validate the RNA-seq data by testing the differential expression of a few miRNAs detected by RNA-seq in HC samples. To reliably detect the changes identified in RNA-seq only miRNAs upregulated in individual compartments were selected that were highly expressed and significantly deregulated. Selected miRNAs upregulated in the RNA-seq data were also significantly enriched in the nucleus in RT-qPCR analysis (Supplementary Fig. 4a). Vice versa, enhanced expressions of miRNAs altered in cytoplasmic fractions in RNA-seq data were also detected as upregulated in the cytoplasm by RT-qPCR (Supplementary Fig. 4b).

Together, these data show distinct and converging changes in the sub-cellular expression of miRNAs and isomiRs in different brain regions in mTLE patients.

### Cell type-specific changes in the sub-cellular localization of *miR-92b* in mTLE

To select miRNA candidates for more in-depth analysis of changes in sub-cellular RNA expression, we analyzed the HC RNA-seq data for upregulated miRNAs with a higher mean average of reads per kilobase per million (RPKM) values and FDR < 0.05. Such selection will lead to the identification of the most highly expressed and significantly deregulated miRNAs, which can be most reliably detected and targeted in subsequent experiments. The selection identified *hsa-miR-92b-3p* (miRbase ID: MIMAT0003218) and *hsa-miR-409-3p* (miRbase ID: MIMAT0001639) as top hits for the nucleus and cytoplasm, respectively. In subsequent experiments, we largely focused on *hsa-miR-92b-3p* because of the atypical nuclear enrichment of this miRNA. This nuclear enrichment was first confirmed by ISH using LNA probes in an independent set of patient tissue samples (Fig. 2g–h). Strong nuclear expression of *miR-92b-3p (miR-92b)* was present in the CA1 and CA3 regions of the hippocampus compared to control hippocampus (Fig. 2g). Interestingly, neurons in the dentate gyrus did not display increased nuclear localization of *miR-92b* (Fig. 2g). Similarly, the sub-cellular localization of *miR-92b* was normal (mainly cytoplasmic) in control cortical tissue or in cortical tissue removed to access an underlying brain tumor (Fig. 2h)*.* These observations confirm and extend our previous work showing nuclear enrichment of *miR-92b* in human mTLE non-HS and mTLE + HS HC samples [56]. Finally, expression of another miRNA with predicted increased nuclear expression, *miR-423-3p*, was examined by LNA-ISH confirming that altered nuclear miRNA localization in mTLE is not restricted to *miR-92b* (Supplementary Fig. 4c). Overall, these results identify cell type-specific nuclear enrichment of *miR-92b* in the HC in the absence of overt changes in its cytoplasmic levels.

### *miR-92b* binding partners in the nucleus link to ribosomal function and regulation

**Fig. 3:**
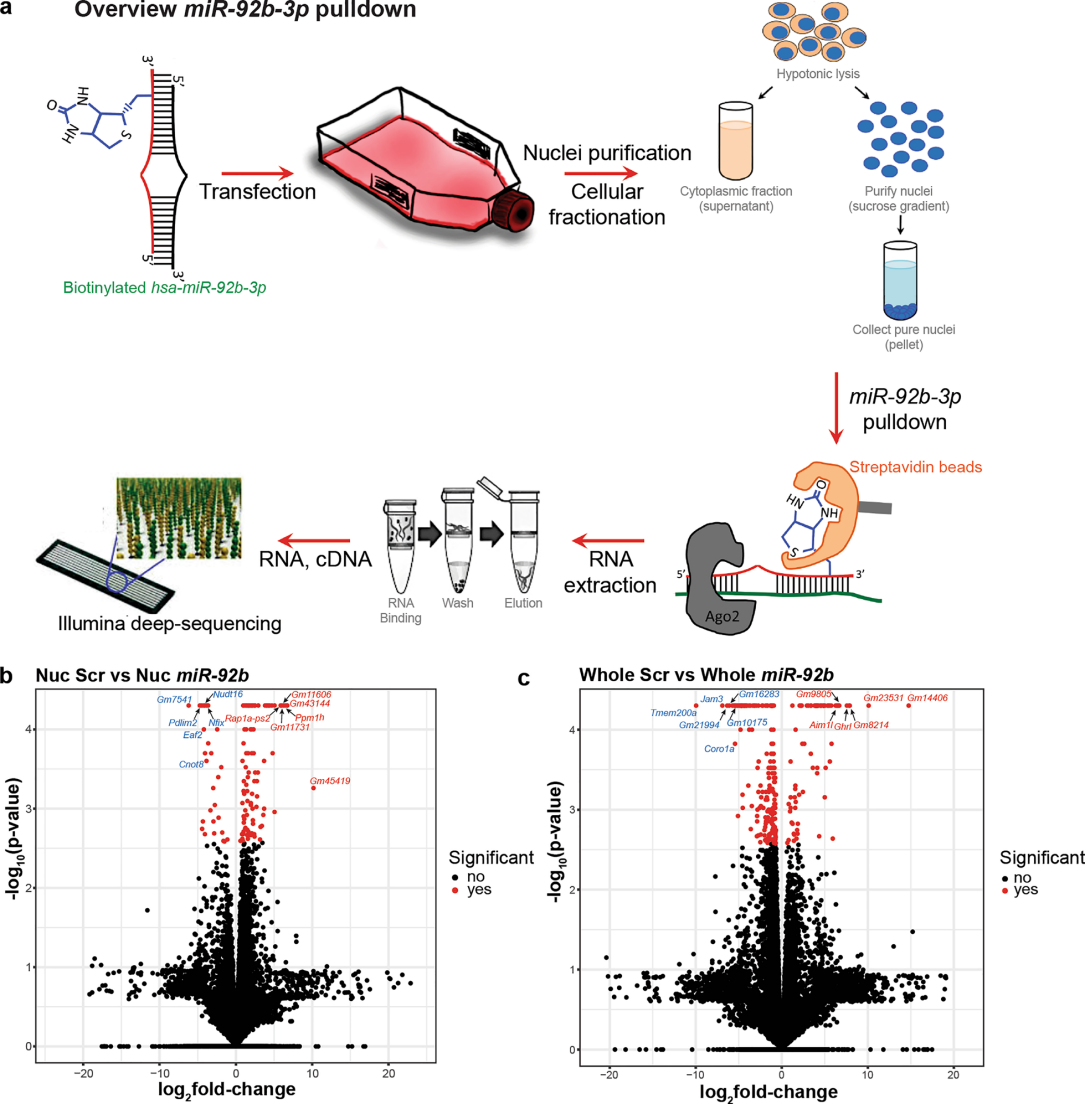
Identification of *miR-92b*-binding partners in neuronal cells. **a** Schematic illustrating the methods used to pulldown biotinylated *miR-92b* from Neuro2A cells (nucleus or whole cell) followed by RNA-seq. Ago2, argonaut2. **b** Volcano plots showing the DE genes following *miR-92b* pulldown from nuclear (Nuc) or whole cell (whole) Neuro2A samples. *n* = 3 samples per group, significantly DE genes are shown in red (padj < 0.05; logFC > 1 and < -1). A selection of strongly upregulated (in red) or downregulated (in blue) genes are indicated in the plots. For DE transcripts, see Supplementary excel file (Tab25 and Tab26)

*miR-92b* is part of the *miR-25* family of miRNAs and is abundantly expressed in several human tissues, with strong expression in brain, nerves, and spinal cord (human tissue atlas; [60]). *miR-92b* has different neural functions, including regulation of intermediate cortical progenitors, promotion of spinal cord regeneration, and protection of neurons from oxygen and glucose deprivation [18, 54, 133]. To further study the nuclear enrichment of *miR-92b*, we performed pulldown assays for biotin-tagged *miR-92b* in nuclear and whole cell, as a control, lysates from mouse neuronal [Neuro2A (N2A)] cells 48 h after transfection (Fig. 3a). The purity of the different fractions was confirmed at the protein and RNA level (Supplementary Fig. 5a, b). Biotin-tagged *miR-92b* efficiently localized to the cell nucleus, as shown by increased expression in the nucleus after fractionation (Supplementary Fig. 5c), and mimicked the increased expression observed in human mTLE nuclei. Identification of nuclear *miR-92b*-binding partners and their associated pathways could help dissect the (novel) functions of *miR-92b* in the nucleus in mTLE. Streptavidin-coated beads were used to isolate biotin-tagged *miR-92b* and binding partners from nuclear and whole cell lysates followed by RNA-seq (Fig. 3a). PCA of the RNA-seq data revealed clear separation between nuclear and whole cell samples supporting the existence of a nucleus-specific RNA interactome for *miR-92b* that differs in part from interactions found at the whole cell level. Although the segregation between groups (*miR-92b* versus scrambled control) was not clear (Supplementary Fig. 5d), several differentially expressed genes were detected in biotin-*miR-92b* nuclear and whole cell samples as compared to scrambled controls (129 and 267 genes for nuclear and whole cell samples, respectively) [Fig. 3b,c; Supplementary excel file (Tab25 and Tab26)]. Panther overrepresentation analysis of the differentially expressed genes identified different pathways in the nuclear fractions, including eukaryotic translational elongation, protein methylation, ribosomal protein formation, and regulation and mitochondrial-related pathways as respiratory electron transport and complex I biogenesis [Supplementary excel file (Tab27)]. In whole cell samples, pathways, such as ER quality control compartment, translational initiation complex formation, and regulation of PTEN localization, were most significantly enriched [Supplementary excel file (Tab28)]. The pathways that were most enriched in both samples were those for ribosomal protein formation and function. This is interesting as genes encoding small nucleolar RNAs (snoRNAs), another class of small ncRNA, were differentially expressed in nuclear and whole cell biotin*-miR-92b* IP samples as compared to scrambled control [Supplementary excel file (Tab25 and Tab26)]. SnoRNAs localize to the nucleolus and have roles in RNA modification and pre-ribosomal RNA processing [51].

Thus, in the nucleus of neuronal cells, a subset of *miR-92b* RNA-binding partners function in ribosomal function and regulation, including snoRNAs.

### Accumulation of *miR-92b* in the neuronal nucleolus in human and experimental mTLE

**Fig. 4:**
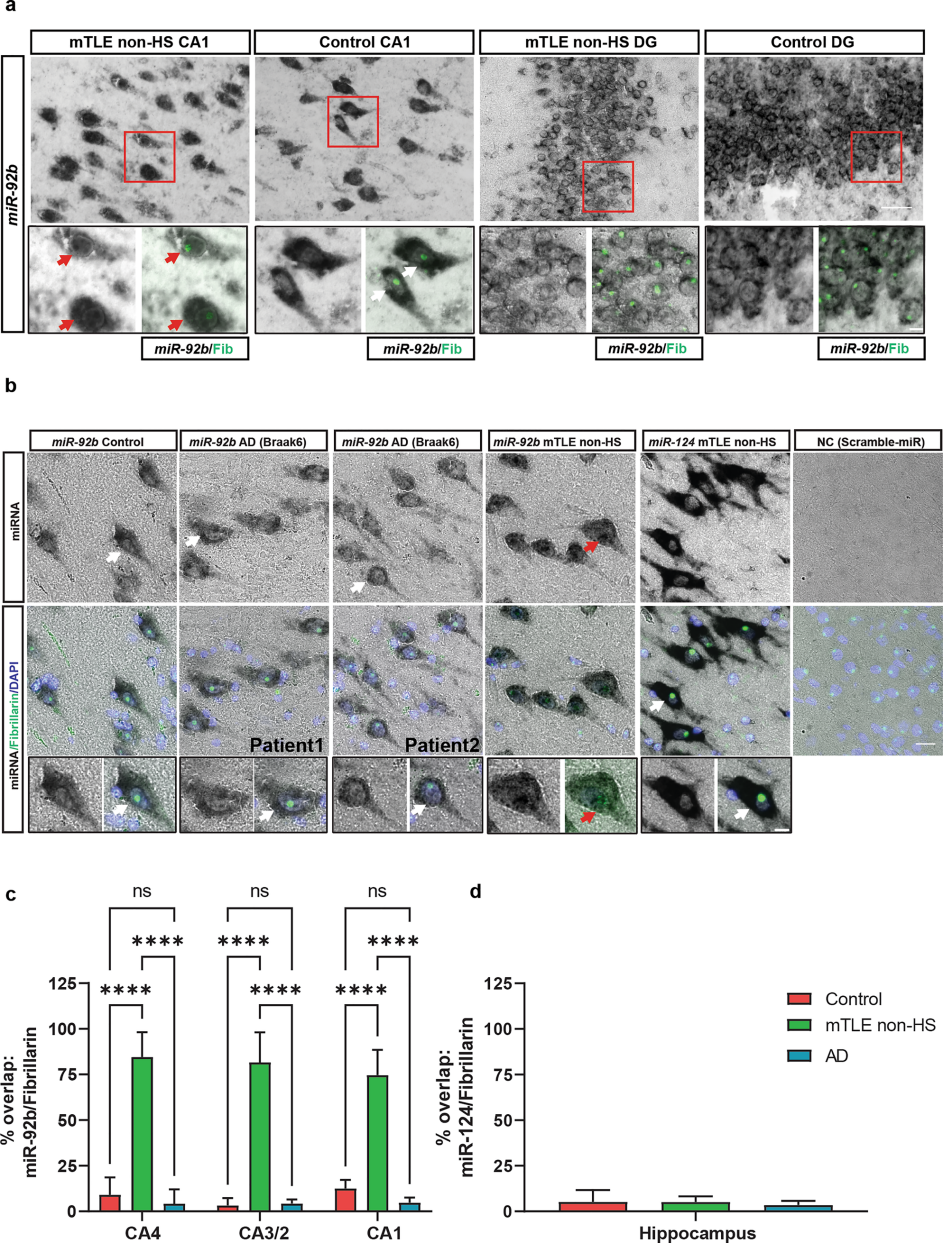
Nucleolar enrichment of *miR-92b* in hippocampal neurons in human mTLE. **a** Representative images showing in situ hybridization (ISH) for *miR-92b-3p* in the CA1 and dentate gyrus (DG) regions of the hippocampus of control and mTLE non-HS cases in combination with immunohistochemistry for fibrillarin (Fib, in green; lower right panel). Boxed area is shown at higher magnification in the lower two panels. Nuclear signals and overlap with Fib staining are only observed for mTLE CA1 neurons (red arrows). White arrows indicate lack of enriched nuclear *miR-92b* signals. *n* = 2 ISH experiments were performed with similar results. *n* = 3 control, *n* = 4 mTLE non-HS. Scale bar, 50 μm and 10 μm (inset). **b** Representative images of *miR-92b* or *miR-124* ISH in postmortem control, AD (2 different patients) and mTLE non-HS HC tissue (CA region) in combination with immunohistochemistry for Fib and DAPI (lower panels). NC, scrambled control probe. White arrows indicate lack of enriched nuclear *miR-92b* and *miR-124* signals. Red arrow indicates nuclear accumulation of *miR-92b*. Scrambled control probe did not yield specific signals. Scale bar, 20 μm and 10 μm (inset). *n* = 2 ISH experiments were performed with similar results. *n* = 3 control, *n* = 4 mTLE non-HS, *n* = 5 AD. **c** Quantification of the percentage overlap of the *miR-92b* ISH and Fib IHC signals in different HC sub-regions (CA4, CA3/2, and CA1). Data (cells) are shown as means + SD. Two-way ANOVA, ****p < 0.001, ns, not significant. n (cells counted) = 817, 517 and 889 in controls (*n* = 4), mTLE non-HS (*n* = 4), and AD (*n* = 5) tissue, respectively. **d** Quantification of the percentage overlap of the *miR-124* ISH and Fib IHC signals in the HC. One-way ANOVA, ns. *n* (cells counted) = 263, 334, and 386 in controls (*n* = 3), mTLE non-HS (*n* = 3), and AD (*n* = 3), respectively.

Given the identification of snoRNAs as potential binding partners of *miR-92b* in the nucleus and their nucleolar localization, we carefully assessed the nuclear localization of *miR-92b* in mTLE. LNA-ISH showed that although *miR-92b* signals are present in the nucleus of HC neurons in control and mTLE tissues, *miR-92b* only accumulated in small structures in the nucleus of human mTLE non-HS but not control HC tissue. These structures were positive for the nucleolus marker FBRL (Fig. 4a). In comparison, *miR-124-3p*, a neuron-enriched miRNA, was localized specifically to the cytoplasm in human mTLE non-HS HC tissue (Supplementary Fig. 6a–d). In addition, in line with our data on the strong nuclear expression of *miR-92b* (Fig. 2e), nucleolar enrichment was present in the CA but not DG region in mTLE non-HS (Fig. 4a; insets). As DG granule cell dispersion is found in mTLE + HS but not mTLE non-HS tissue (Blumcke et al., 2013), we also examined mTLE + HS HC but found no nucleolar enrichment of *miR-92b* in DG granule cells in these patients (Supplementary Fig. 7).

Next, we checked if the nucleolar accumulation of *miR-92b* is specific to mTLE or can also be observed in other diseases in which the HC is affected, such as Alzheimer’s disease (AD). However, although *miR-92b* signals were detected in nuclei of HC neurons in Braak6 AD patient tissue, no specific enrichment in FBRL-positive nucleoli was found (Fig. 4b). Quantification of the LNA-ISH data confirmed our qualitative observations and showed strongly increased co-localization of *miR-92b* and FBRL in neurons in the HC CA regions in mTLE non-HS but not AD patient tissue (Fig. 4c). In addition, co-localization for *miR-124* and FBRL was almost absent in mTLE and AD (Fig. 4d).

Finally, to assess whether the nucleolar accumulation of *miR-92b* is characteristic of chronic mTLE stages (such as in resected human brain tissue) or also present at early disease stages, we performed LNA-ISH for *miR-92b* on HC tissue from a pilocarpine rat model of TLE at different timepoints after SE induction (2, 4, 8, and 19 weeks) (Supplementary Fig. 8a). This model has a latent period without seizures until 14 weeks after SE and from 15 weeks onwards spontaneous recurrent seizures (SRS) are observed [57, 125]. Interestingly, robust nucleolar *miR-92b* accumulation was found in CA neurons after SE as compared to control rats (Supplementary Fig. 8b). Quantification of *miR-92b* signal in the nucleus confirmed this result and showed a significant increase at 2, 8, and 19 weeks after SE compared to controls (Supplementary Fig. 8c, d). However, while human neurons showed enrichment of both nuclear and nucleolar *miR-92b*, nuclear accumulation of this miRNA was mostly restricted to the nucleolus in the experimental rat model. To examine whether the nucleolar enrichment of *miR-92b* is a general feature of epilepsy, we performed ISH for *miR-92b* in mice carrying a *tuberous sclerosis complex 1* (*Tsc1*) gene deletion. TSC patients with brain lesions develop severe seizures and two-thirds of these patients do not respond to available AED therapies [22, 24, 92]. *Tsc1* knockout mice show activation of the mammalian Target of Rapamycin Complex I (mTORC1) pathway resulting in severe seizures and death [2, 66]. In contrast to our observations in human and experimental (rat pilocarpine) TLE, no specific nucleolar enrichment was observed in *Tsc1-cre*^+^, as compared to *Tsc1-cre*^*−*^*,* mouse hippocampal neurons (Supplementary Fig. 9a).

Together, these results show that *miR-92b* accumulates in the nucleolus of specific HC neurons during epileptogenesis leading to TLE.

### Changes in nucleolar morphology and the distribution of key nucleolar proteins in mTLE

**Fig. 5:**
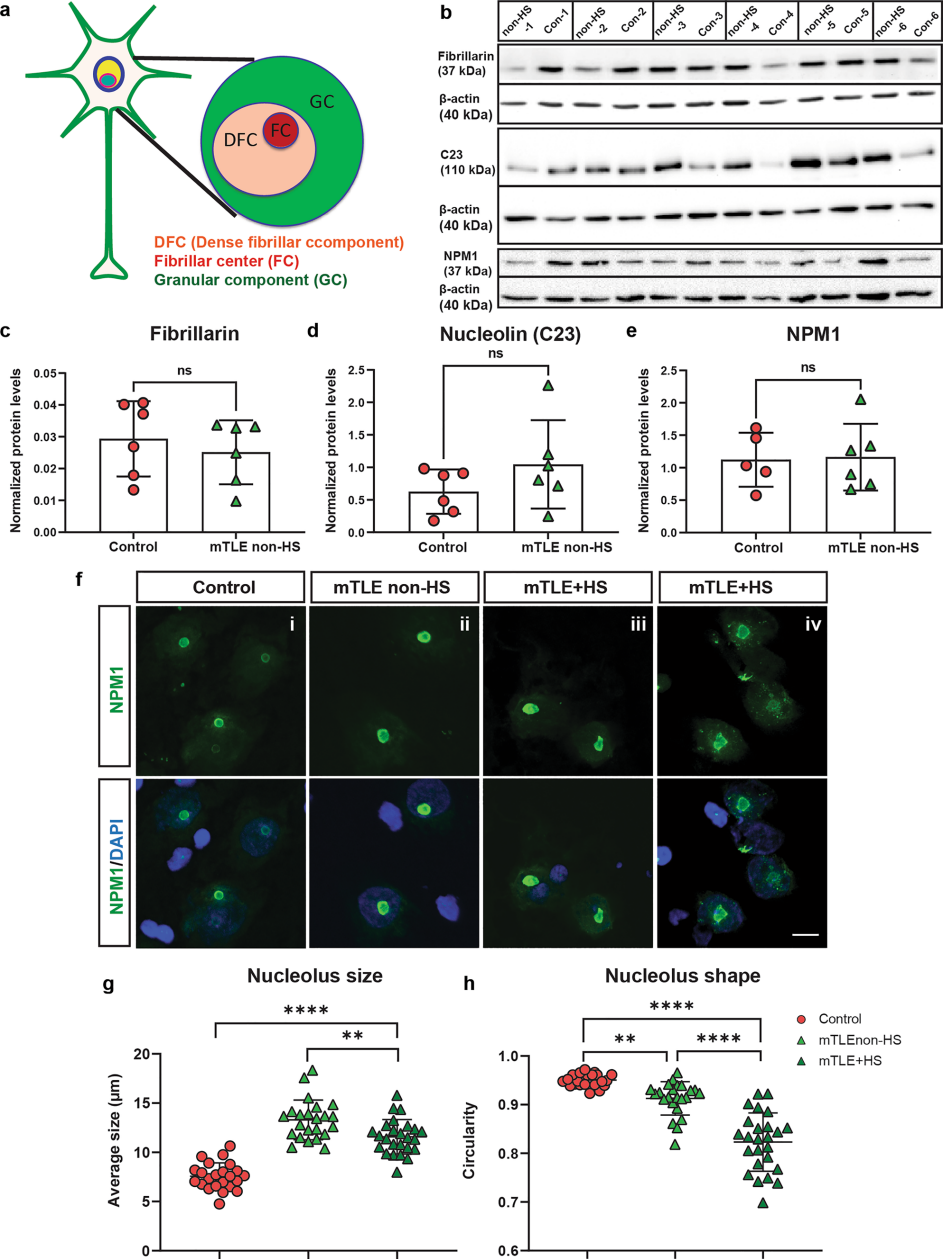
Changes in the size and shape of neuronal nucleoli in human mTLE. **a** Schematic showing the different sub-regions of a neuronal nucleolus. Fibrillar center (FC), granular component (GC), and dense fibrillar center (DFC). Fibrillarin marks the FC, and NPM1 and C23 antibody the GC. **b** Western Blot analysis of control and mTLE non-HS hippocampus (HC) tissue (*n* = 6 control and mTLE non-HS cases) for fibrillarin, NPM1, C23, and *β*-actin (loading control). **c**–**e** Quantification of western blots as in **b** showing normalized protein levels (to *β*-actin) for fibrillarin (**c**), C23 (**d**), and NPM1 (**e**) for control and mTLE non-HS HC tissue. *n* = 6 controls and mTLE non-HS. For NPM1, *n* = 5 control and 6 patient samples were used (Con-5 was excluded from analysis due to an air bubble in the band). Mann–Whitney test. *ns*, not significant. Data (cases) are shown as means ± SD.** f** Representative images of immunohistochemistry for NPM1 in sections of the HC CA region of control (i) and mTLE non-HS (ii), and mTLE + HS (iii–iv) cases. DAPI in blue. Both the size and shape of NPM1 nucleoli is altered in mTLE. Scale bar, 10 μm. **g** Quantification of nucleolus size based on images as in **f**. Each datapoint represents mean size estimated of all measured cells determined from each individual images (control 23 images, mTLE non-HS 23 images, and mTLE + HS 25 images with at least 5 images per individual) collected from *n* = 4 controls and *n* = 4 mTLE patients. Ordinary one-way ANOVA with Sidak’s multiple comparisons, ****p < 0.0001, **p < 0.005. **h** Quantification of nucleolus shape based on images as in **f**. Each datapoint represents mean shape estimated of all measured cells determined from individual images (control 23 images, mTLE non-HS 23 images, mTLE + HS 25 images with at least 5 images per individual) collected from *n* = 4 controls and *n* = 4 mTLE patients. Shape was assessed as circularity with values closer to 1 being more circular. Ordinary one-way ANOVA with Sidak’s multiple comparisons, ****p < 0.0001, **p < 0.005

The accumulation of *miR-92b* in the nucleolus in mTLE prompted us to characterize this structure in more detail. The nucleolus is a membrane-less substructure of the nucleus, which exists as a phase-separated unit from the rest of the nucleus. The different components of nucleolus include: (1) the fibrillar center (FC), where rRNA transcription occurs, and (2) the dense fibrillarin component (DFC) and granule component (GC) where subsequent steps of rRNA processing and ribosome assembly happen. Then, these assembled ribosome subunits are transported to cytoplasm to form ribosomes and aid in protein translation [47]. Nucleolar stress caused by accumulation of mis-folded proteins is observed in several neurodegenerative diseases [69, 93, 137]. Further, the size and shape of nucleolus are linked to proper functioning of this structure [136]. Therefore, we studied the morphology of nucleoli in mTLE HC using key nucleolar proteins as markers: FBRL, a protein localized to DFC, and nucleophosmin (NPM1) and Nucleolin (C23), which localize to the GC (Fig. 5a). Western blot analysis of HC tissue for FBRL, C23, and NPM1 did not reveal differences in the overall expression of these proteins between mTLE non-HS and control (Fig. 5b–e). However, immunohistochemistry for NPM1, which marks the GC, revealed an increase in the size of neuronal nucleoli in HC tissue of both mTLE non-HS and mTLE + HS patients compared to control (Fig. 5f(i–iii)–g). Enlarged neuronal nucleoli were also found in the CA1 and CA2 regions of the pilocarpine rat model, as early as 2 weeks after SE induction. At 19 weeks, the increase was observed in all three CA regions (CA1, CA2, and CA3) of the hippocampus (Supplementary Fig. 9b–d). In addition to size changes, we also detected an altered nucleolar shape in mTLE non-HS and mTLE + HS HC tissue, with a stronger effect in mTLE + HS patients (Fig. 5f(iv), h). NPM1 fluorescence intensity was not altered between control and mTLE conditions confirming the Western blot results (Supplementary Fig. 9e).

**Fig. 6:**
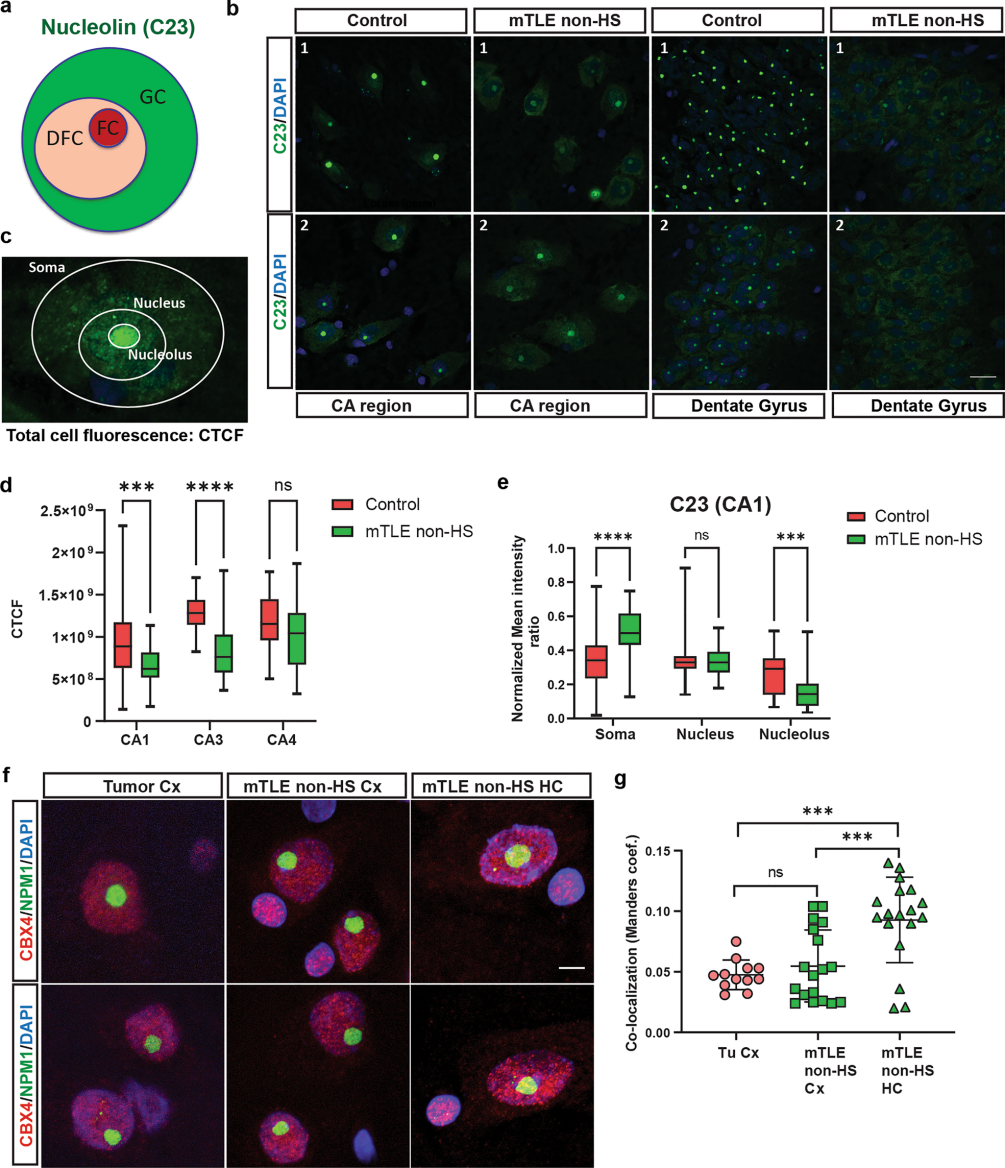
Abnormal localization of C23 and CBX4 in human mTLE hippocampus. **a** Schematic depicting key nucleolar regions labeled in different colors. Fibrillar center (FC), granular component (GC), and dense fibrillar center (DFC). C23 (nucleolin) marks the GC. **b** Representative images of immunohistochemistry for C23 in sections of the HC region [CA and dentate gyrus (DG)] from control and mTLE non-HS cases (*n* = 2 each). DAPI in blue. Scale bar, 20 μm. **c** Image showing C23 immunosignal and labeled for different individual sub-cellular compartments in which C23 immunofluorescence was measured. **d** Boxplots showing quantification of CTCF (total cell fluorescence) of C23 immunostaining in different CA hippocampal regions in control and mTLE non-HS cases from images as in **b**. C23 signals were reduced in the CA1 and CA3 regions. ANOVA with Sidak’s multiple comparisons test; ****p < 0.0001, ***p = 0.0009. n (cells measured) = CA1 54 from Controls and mTLE non-HS; CA3 26 and 36 in control and mTLE non-HS, respectively; CA4 28 and 29 cells from controls and mTLE non-HS, respectively, and from *n* = 4 control and *n* = 3 mTLE non-HS cases. **e** Boxplots showing quantification of C23 immunofluorescence in neuronal soma, nucleus, and nucleolus in the CA1 hippocampal region in control and mTLE non-HS cases from images as in **b**. C23 signals in the soma were increased and nucleolar labeling decreased in mTLE compared to control. Fluorescence signal in the nucleus was unchanged. ANOVA with Sidak’s multiple comparisons test; ns, not significant. ****p < 0.0001, ***p = 0.0001. *n* (cells measured) = CA1 54 each from Controls and mTLE non-HS from *n* = 4 control and *n* = 3 mTLE non-HS cases. **f** Representative images of double immunohistochemistry for NPM1 and CBX4 in sections of the Cx and HC from tumor control (Cx) and mTLE non-HS cases (Cx and HC). DAPI in blue. Scale bar, 5 μm. **g** Quantification of the co-localization of NPM1 and CBX4 in images as in **f**. Mander’s coefficient was determined using the JACoP plugin. Each datapoint represents the estimated coefficient determined from individual images collected from *n* = 2 tumor control (Tu Cx), *n* = 3 mTLE-non-HS Cx, and *n* = 3 mTLE non-HS HC. Data are shown as means ± SD. ANOVA with Tukey’s multiple comparisons test. ns, not significant, ***p < 0.001.

To confirm the nucleolar changes observed using NPM1 immunostaining, we analyzed an additional nucleolus marker, C23. C23 is an RNA-binding protein mainly localized to the GC of nucleoli, but able to shuttle between nucleus and cytoplasm in healthy and disease conditions (Fig. 6a) [1]. In control tissue, C23 was detected in the nucleolus. In the mTLE non-HS HC reduced nucleolar C23 staining was observed together with increased signals in the soma compartment in both CA and DG regions (Fig. 6b). Quantification confirmed an overall reduced fluorescence intensity for C23 in CA1 and CA3 region but not CA4 region in mTLE non-HS (Fig. 6c, d). Further, increased C23 expression was found in the soma compartment and reduced levels in the nucleolus in mTLE non-HS as compared to control. Signal in the nucleus was unaltered, hinting at a redistribution of C23 from nucleoli to the soma compartment (Fig. 6e; Supplementary Fig. 10a–b). Quantification of nucleolus size based on C23 labeling revealed an increase in the size of nucleoli in the different CA regions in the absence of changes in the size of the nucleus (Supplementary Fig. 10c–d), in line with the NPM1 analysis (Fig. 5f–g).

Nucleoli in eukaryotic cells function as phase-separated protein quality control compartments, and during cellular stress, several proteins localize to this structure to be stored until stress is relieved or to be targeted for degradation [4, 38]. We therefore assessed whether the expression and localization of the polycomb repressive complex proteins CBX4 and CBX8, two other proteins known to localize to the nucleolus, are altered in mTLE. Total HC levels of CBX4 and CBX8 were similar for control and mTLE non-HS tissue (Supplementary Fig. 10e–g). To assess the sub-cellular distribution of CBX proteins, immunostaining for CBX4 was performed. Here, we used cortical tissue that was removed during tumor resection as a control as we were unable to get specific signals for CBX4 in postmortem tissues. Immunohistochemistry for CBX8 was in general unsuccessful. Under normal conditions, CBX4 is localized to the polycomb regions in the nucleus avoiding the nucleolar compartment, which we observed in Cx control and mTLE non-HS tissue. However, in mTLE non-HS HC tissue co-localization of CBX4 with NPM1 into the nucleolus was found (Fig. 6f). Quantification of CBX4 and NPM1 co-localization confirmed these results (Fig. 6g).

Overall, these results indicate that nucleoli in mTLE HC display changes in nucleolar size and shape, and in the redistribution of key nuclear protein into and out of the nucleolus, which are typical signs of cellular stress.

### SnoRNA are deregulated in mTLE

**Fig. 7:**
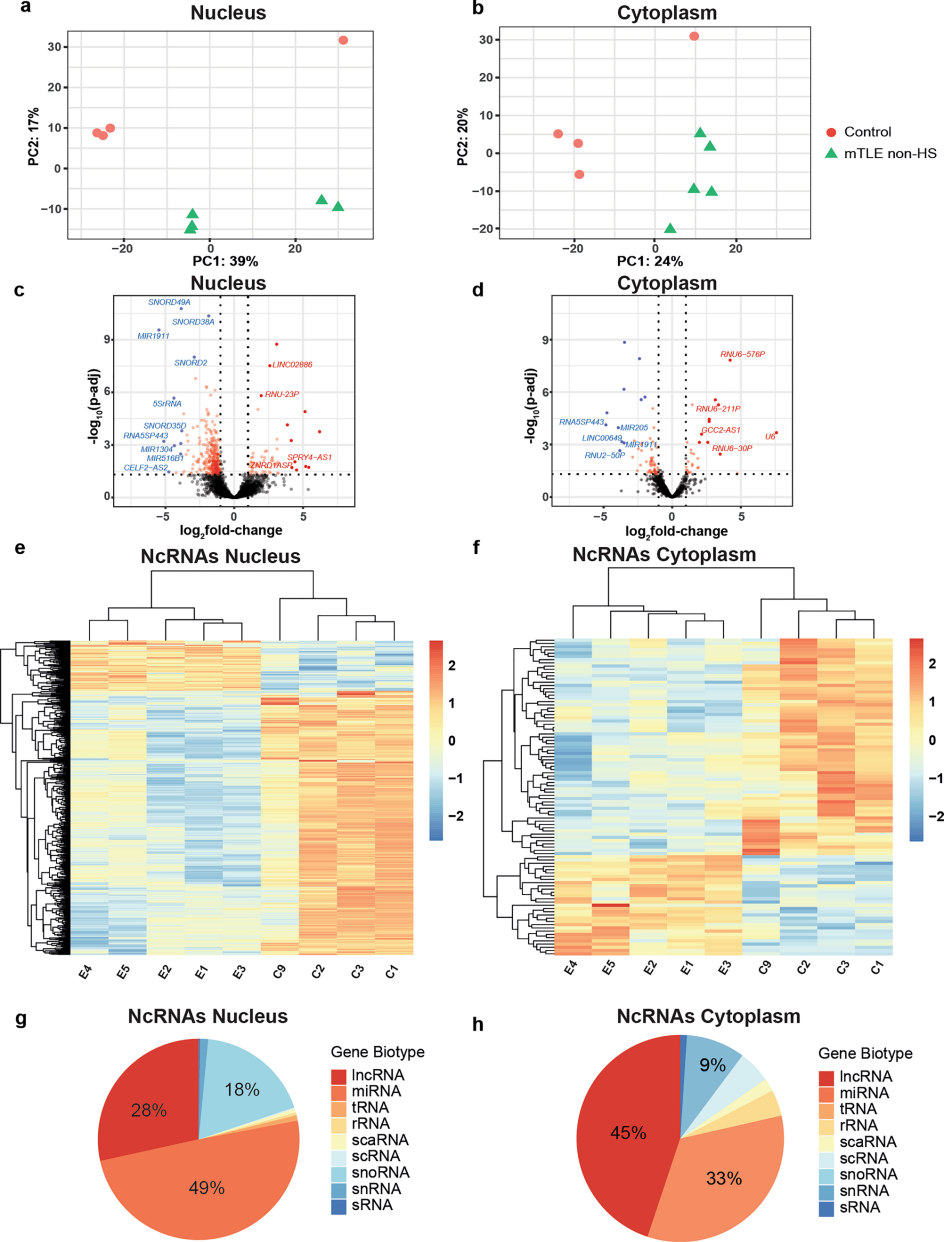
RNA seq analysis of human mTLE hippocampal cell compartments for all ncRNAs. **a, b** Principal Component Analysis (PCA) for hippocampal (HC) nucleus and cytoplasmic fractions from controls (*n* = 4) and mTLE non-HS patients (*n* = 5) based on deregulated small non-coding RNA (ncRNA) expression detected by small RNA-seq. **c, d** Volcano plots showing DE ncRNAs from nucleus and cytoplasm fractions in mTLE non-HS (as compared to control). Significantly DE genes are shown in red (pad*j* < 0.05; logFC > 1 and <  − 1 [representing at least one fold change of difference in expression compared to control samples)]. A selection of strongly upregulated (in red) or downregulated (in blue) genes are indicated in the plots. For DE ncRNAs, see Supplementary excel file (Tab19 and Tab20). **e, f** Heatmap showing differentially expressed small ncRNAs in hippocampal (HC) nucleus and cytoplasmic fractions from controls (*n* = 4; C1–C3, C9) and mTLE non-HS patients (*n* = 5; E1–E5). For DE ncRNAs see Supplementary excel file (Tab19 and Tab20). **g, h** Pie chart showing the contribution of different classes of ncRNAs in the list of DE ncRNAs for nuclear (**g**) and cytoplasmic (**h**) fractions.in mTLE non-HS (as compared to control)

The changes observed in nucleolar morphology and protein distribution hint at a loss of nucleolar integrity. This could cause increased localization of RNAs to the nucleolus, such as *miR-92b*, but also changes in RNAs that reside and function in the nucleolus. Interestingly, *miR-92b* pull down from the nucleus identified snoRNAs (Fig. 3). SnoRNAs are essential, nucleolus-localized RNAs with main functions in 2-*O*’-methylation (box C/D) and pseudouridylation (box H/ACA) of ribosomal RNA (rRNA), small nuclear RNAs (snRNA), and other classes of RNAs [9, 25, 34]. To examine whether the expression of snoRNAs and other non-coding RNAs is altered in mTLE, we analyzed the small RNA-seq data from mTLE non-HS HC cytoplasmic and nuclear fractions. Reads were controlled for quality and followed up with quasi-mapping and DEseq2 processing (Supplementary Fig. 11a). To improve mapping rates and facilitate detection of the majority of ncRNA classes, including snoRNAs, k-mer size of different lengths was optimized [19]. K-mer 13 showed a high mapping rate [Supplementary excel file (Tab18)]. Next, we tested our pipeline of snoRNA analysis with published datasets and obtained similar mapping percentages (74.7% and 74.1% respectively; GSE140623; [77]). Small RNA-seq data from mTLE nuclear and cytoplasmic samples was mapped with human ncRNA index using Salmon [96], which provides data on snoRNAs and other classes of ncRNAs. The average percentage of assigned ncRNA reads obtained for mTLE non-HS and control samples was between 60 and 80% [Supplementary Fig. 11b; Supplementary excel file (Tab18)]. Finally, DEseq2 was used to perform differential gene expression analysis, showing a lower predicted dispersion with a higher mean of normalized counts (Supplementary Fig. 11c). Principal component analysis (PCA) showed clear separation between sample clusters from patients and controls, and for nuclear and cytoplasmic fractions (Fig. 7a, b). The majority of differentially expressed genes were downregulated (Fig. 7c, d; Supplementary Fig. 11d). Samples belonging to the same patient group clustered together and showed similar expression levels for the different genes (Fig. 7e, f). From all transcripts, cytoplasmic fractions showed 98 DE genes and nuclear fractions with 464 DE genes. In the nucleus, most of the differentially expressed genes were miRNAs followed by long non-coding RNAs and snoRNAs. In the cytoplasm, lncRNAs were most abundantly deregulated followed by miRNAs [Fig. 7g–h; Supplementary excel file (Tab19 and Tab20)].

**Fig. 8:**
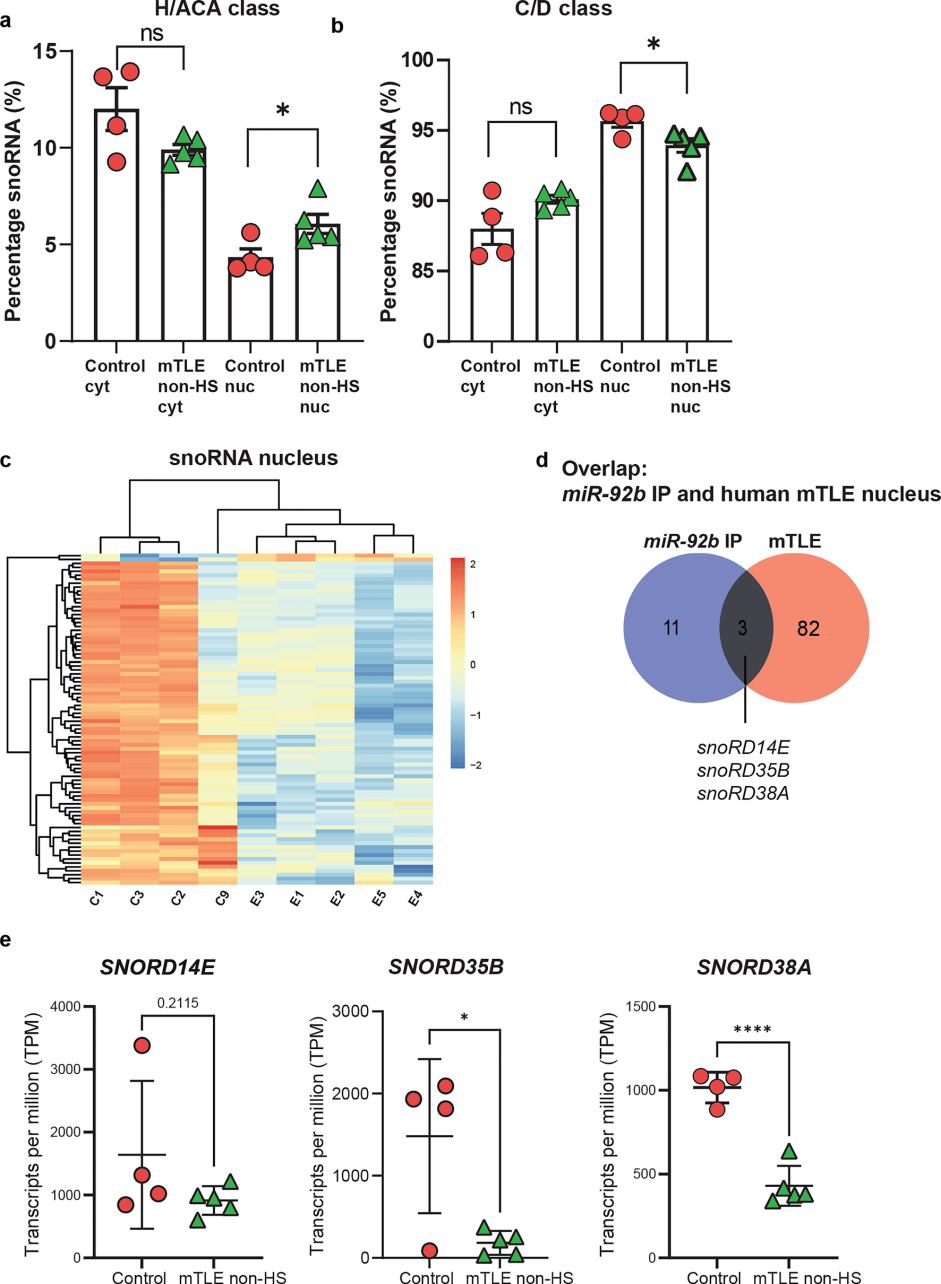
Differential snoRNA expression in human mTLE brain tissue. **a**, **b** Percentage of assigned reads for H/ACA and C/D class snoRNAs in cytoplasmic (cyt) and nuclear (nuc) fractions from control and mTLE non-HS HC samples. *n* = 4 control and *n* = 5 mTLE non-HS cases. Data are means ± SD. Unpaired t test, *p < 0.05, ns, not significant. **c** Heatmap showing differentially expressed snoRNAs in nuclear fractions from mTLE non-HS HC samples. Controls (*n* = 4; C1–C3, C9) and mTLE non-HS patients (*n* = 5; E1–E5). For DE snoRNA list, see Supplementary excel file (Tab20). **d** Venn diagram showing the overlap of snoRNAs identified in *miR-92b* immunoprecipitation and differentially expressed snoRNAs in nuclear fractions from mTLE non-HS HC. **e** Normalized transcripts per million (TPM) counts of the three common DE snoRNAs (see **d**) in the nucleus of control and mTLE non-HS cases. *n* = 4 control and *n* = 5 mTLE non-HS cases. Data are means ± SD. Unpaired t test; *p < 0.05; ****p < 0.0001

This analysis confirmed prominent differential expression of snoRNAs in nuclear fractions from mTLE tissue. Interestingly, class-specific changes were observed with an increase in H/ACA class snoRNAs in nuclear mTLE non-HS fractions as compared to control and reduced expression of C/D class snoRNAs (Fig. 8a, b). Such differences could be from altered biogenesis due to the nucleolar stress mTLE tissue. In line with the fact that most snoRNAs localize to the nucleolus, 85 differentially expressed snoRNAs were found in nuclear fractions and only 4 in the cytoplasm. The majority of the snoRNAs were downregulated in mTLE except a few that were upregulated (Fig. 8c, Supplementary Fig. 12a). Interestingly, a few nuclear downregulated C/D class snoRNAs were also identified as binding partners of *miR-92b* (Fig. 3): *snoRD14E*, *snoRD35B*, and *snoRD38A* (Fig. 8d–e, Supplementary Fig. 12b). To begin to understand whether altered expression of snoRNAs can be linked to gene expression changes in mTLE, we estimated the non-ribosomal methylation targets of *snoRD14E*, *snoRD35B*, and *snoRD38A* [59]. Several genes differentially expressed in human TLE samples [(Xin actin-binding repeat containing 1 (*XIRP1*), CC Chemokine ligand 2 (*CCL2*), and Neuronal PAS domain protein 4 (*NPAS4*)] were predicted to have potential methylation sites ([122]; Supplementary Table 4, Supplementary excel file (Tab22 to Tab24)) and showed inverse expression changes to those found for snoRNAs in human TLE. Thus, these results show class-specific changes in snoRNA expression specific sub-cellular compartments in mTLE.

Nucleolar stress is associated with re-localization or depletion of NPM1 from the nucleolus, which can (in)directly affect nucleolar integrity and function [91, 135]. Further, NPM1 acts as a key regulator of *rRNA* 2’-*O*-methylation by directly binding to *C*/*D* class snoRNAs [87]. Interestingly, our data show an altered nuclear distribution of NPM1 and deregulation of *C*/*D* class snoRNAs (*snord14e*, *snord35a*, *snord38a*) in TLE. Therefore, as a first step to understand the role of NPM1 in TLE, we examined whether deregulation of NPM1 affects the *C*/*D* class snoRNAs found to be altered in TLE. We exploited the CRISPR-Cas13 system to induce NPM1 knockdown in mouse neuronal cells. From three guide RNAs designed and tested, one induced significant knockdown of NPM1 in N2A cells in vitro (50% reduction at the RNA level detected by RT-qPCR and 40% reduction at the protein level detected by Western blotting) (Supplementary Fig. 12c–e). Next, NPM1 knockdown was induced followed by expression analysis of the three candidate snoRNAs. This revealed a significant increase in the expression of *snord14e* and *snord35a* (Supplementary Fig. 12f, g), and a trend toward increased expression for *snord38a* (Supplementary Fig. 12h). Although future studies are needed to further dissect the precise role of NPM1 and snoRNA deregulation in TLE, our results support the exciting possibility that deregulation of NPM1 may induce changes in snoRNAs in TLE.

## Discussion

Molecular and cellular changes that occur at the sub-cellular level in the brain of mTLE patients remain incompletely understood. Therefore, we established cell compartment-specific small non-coding RNA profiles by performing small RNA-seq on cytoplasmic and nuclear cellular fractions from human brain tissue. This revealed an altered sub-cellular distribution of miRNAs and isomiRs, and reduced levels of snoRNAs. The disease-associated enriched localization of one of the deregulated miRNAs, *miR-92b*, in neuronal nucleoli and its ability to bind snoRNAs, triggered a detailed analysis of neuronal nucleoli in mTLE brain tissue. This uncovered several defects, such as altered nucleolar size or shape and mis-localization of nucleolar proteins, indicative of nucleolar stress. Interestingly, nucleolar phenotypes were already detected before spontaneous seizure onset in a rat model of epilepsy, suggesting that nucleolar changes may contribute to the development of seizures and mTLE. These results for the first time implicate nucleolar defects in the pathogenesis of mTLE and provide a framework for further defining the functional consequences of altered sub-cellular RNA profiles in this disease.

### Sub-cellular changes in miRNA expression in mTLE

Previous studies have shown cell compartment-specific localization of miRNAs in neurons in vitro [58, 61], but whether disease conditions change miRNA distribution in neurons in vivo remains largely unknown. To address this question, we determined sub-cellular miRNA profiles from brain tissue derived from mTLE patients and postmortem controls. For many analyses, non-sclerotic (non-HS) HC tissue was used to capture sub-cellular miRNA changes in the absence of the extensive cell death found in HS + mTLE [10]. Indeed, for analyses where HS + tissues were included, changes were often more pronounced in HS + cases (e.g., sub-cellular changes in miRNA expression in Cx). Although postmortem delay may affect RNA levels and distribution, several lines of experimental evidence suggest that this was not causing the compartment-specific changes observed in this study. First, experimental postmortem delay did not induce overt changes in sub-cellular miRNA expression. Second, freshly dissected human control Cx tissue showed a cytoplasmic distribution of *miR-92b* similar to postmortem control. Third, while in freshly dissected control rat HC *miR-92b* was mostly confined to the neuronal cytoplasm, SE induced accumulation of this miRNA in the nucleolus. Fourth, several of the phenotypes observed were stronger or more widespread in HS + as compared to non-HS tissues (e.g., changes in nucleolus shape).

While PCA based on DE miRNA profiles showed clear segregation of HC samples, Cx samples did not segregate well. In line with this, many more DE miRNAs were detected in HC as compared to Cx mTLE tissue. In general, changes were also more abundant in nuclear fractions and only a few DE miRNAs were shared between HC and Cx tissues. Together, these findings indicate that the sub-cellular changes observed are likely in part cell type-dependent. This is also supported by the observation that *miR-92b* accumulated in the nucleus of CA but not DG granule cells in human and experimental TLE. The mechanisms underlying these differences are unknown but may include distinct molecular or cellular features, or proximity to seizure generating foci in refractory mTLE [118].

Changes in posttranscriptional mechanisms are known to contribute to the development and progression of epilepsy [46, 55, 95, 99]. The functional consequences of the sub-cellular changes in miRNA expression in mTLE observed in our study remain unknown, but increased cytoplasmic miRNA levels could increase target inhibition, while nuclear miRNAs may acquire novel targets or act in a non-canonical manner by, for example, binding DNA [48, 50, 140]. Immunoprecipitation of biotinylated *miR-92b* from the nucleus of neuronal cells identified RNAs encoding proteins linked to, e.g., complex I biogenesis, respiratory electron transport, and ribosomal pathways. Since dysfunctional mitochondria and protein synthesis pathways have been implicated in the pathogenesis of epilepsy [98], it is tempting to speculate that altered *miR-92b* expression may affect these processes in mTLE. IsomiR analysis of the DE miRNAs revealed an extra layer of molecular deregulation by unveiling differences in the abundance of specific isomiRs in human mTLE HC. Nucleotide changes in isomiRs, as compared to their canonical miRNA counterpart, can impact miRNA stability or target binding [134]. Further work is needed to establish the molecular and functional consequences of these sub-cellular changes in isomiR expression in mTLE.

### Molecular and morphological changes in the neuronal nucleolus in mTLE

Previous work had shown expression of miRNAs in the nucleolus of non-neuronal cells in vitro, some of which localized to the GC compartment [70, 100]. Our study detects *miR-92b* in the neuronal nucleolus in vivo and shows that disease states can cause miRNA accumulation in the nucleolus. Interestingly, this nucleolar localization is neuron type-specific (observed in CA but not DG neurons). Further, it occurs early during the process of epileptogenesis in experimental models but is not found in human AD HC neurons nor in neurons lacking the *Tsc1* gene, a model mimicking a form of genetic epilepsy [74]. It is unknown why *miR-92b* accumulates in the nucleolus in mTLE, but its nucleolar enrichment could be facilitated by changes in nucleolar protein expression or morphology.

In addition to specific nucleolar perturbations, such as depletion of ribosomal proteins or nucleolar factors, general stressors, including heat shock, nutrient starvation, UV radiation, hypoxia, and viral infection can induce changes in nucleolar morphology [68, 136]. Furthermore, reduced nucleolar size has been linked to reduced ribosomal biogenesis in neurodegenerative disorders such as AD and Parkinson’s disease [5, 8, 47, 94]. We report the occurrence of an increase in nucleolar size and altered nucleolar shape in mTLE HC and experimental TLE tissue. Increases in nucleolar size are often correlated with increased rRNA synthesis, for example in dividing cells in cancer [23, 120]. However, increased nucleolar size was also observed in amyotrophic lateral sclerosis (ALS) patients carrying a *C9ORF72* mutation. In this case, impaired ribosome biogenesis and translation were reported [67, 83]. Therefore, future studies are needed to examine whether the nucleolar changes observed in mTLE reflect nucleolar stress and/or altered ribosomal output. Because of their size and activity, neurons require a high level of rRNA production and protein synthesis [43, 45]. This is even exaggerated in chronic epilepsy where uncontrolled neuronal activity puts further pressure on protein synthesis [62, 102]. Nucleolar changes may not only be induced by these pathogenic situations but could also contribute to their onset or further progression.

In addition to changes in nucleolus size and shape, re-localization of proteins into and out of the nucleolus was observed in mTLE HC. For example, the nucleolar protein C23 was also found in the cytoplasm in mTLE and the polycomb protein CBX4 in the nucleolus. C23 is an RNA-binding protein that primarily localizes to the nucleolus, but under stress conditions can re-locate to either nucleoplasm or cytoplasm [1]. In cytoplasm, C23 binds and regulates the stability and translation of mRNAs [84]. Further, it can interact with nucleic acids that contain G-quadruplex (rG4) structures [108]. Interestingly, the *miR-92b* precursor RNA, *pre-miR-92b*, contains rG4 structures, and C23 and *pre-miR-92b* interactions have been described in vitro [81, 107]. In contrast to C23, CBX4 was present in the nucleolus in mTLE HC tissue, co-localizing with NPM1. Upon stress, nucleoli can function as transient storage locations for mis-folded proteins or target them for degradation under prolonged stress [38]. CBX4 was reported to transiently localize to the nucleolus following heat shock in vitro [4]. As we did not observe changes in overall CBX4 expression, its nucleolar localization most likely does not induce enhanced degradation. However, it could cause a reduced ability of CBX4 to interact with its binding partners outside the nucleolus or act to inhibit excessive rRNA expression, as was reported in mesenchymal stem cells [101].

### Deregulation of snoRNAs in mTLE

Previous studies have shown altered ribosomal function in mouse models of epilepsy and changes in the expression of several ribosomal proteins and snoRNAs in epilepsy patient tissue [80, 98, 126]. Our observations not only confirm but also extend these findings by showing compartment-specific changes in snoRNAs and by revealing that a miRNA that aberrantly accumulates in the nucleolus in mTLE binds snoRNAs in the nucleus. Most DE snoRNAs were downregulated in human mTLE and belonged to the C/D class of snoRNAs. This is in line with the observation that snoRNAs in synaptosomes from hippocampal tissue mainly consist of C/D class snoRNAs [32]. Further, we show that knockdown of NPM1, which shows an altered nuclear distribution in TLE, in neuronal cells causes deregulation of C/D class of snoRNAs shown to be altered in TLE providing a starting point for the future functional analysis of our observations.

Apart from their canonical functions, i.e., 2’-*O*-methylation and pseudouridylation of rRNAs, snoRNAs can regulate the expression of mRNAs, rRNA acetylation, guide tRNA methylation, and modify alternative splicing [13, 28, 35, 49, 53, 112, 127]. Reduced snoRNA levels could therefore have widespread effects. To begin to understand whether altered expression of snoRNAs can be linked to gene expression changes in mTLE, we predicted the 2’-*O*-methylation sites of three snoRNAs that bound *miR-92b* and that showed reduced levels in human TLE HC (*SNORD14E*, *SNORD35B*, and *SNORD38A*). Interestingly, we identified a few target genes previously implicated in epilepsy. Genes differentially expressed in human TLE samples and previously implicated in epilepsy (*XIRP1*, *CCL2*, and *NPAS4*) [15, 39], were predicted to have potential methylation sites and showed inverse expression changes to those found for snoRNAs in human TLE [122]. Although these results suggest that altered snoRNA expression may influence the expression of epilepsy-relevant RNAs, further work is needed to more firmly establish these molecular dependencies and to dissect a potential role for *miR-92b*-snoRNA interactions.

Integration of molecular changes with histopathological alterations has recently been recognized for the classification of brain tumors by the World Health Organization [73]. In line with this, a recent study combined disease-specific DNA methylation signatures with pathological features as an improved classification system for focal cortical dysplasia (FCD) subtypes [65]. Similarly, changes in *miR-92b* localization and snoRNA expression observed in our study could serve as a molecular signature that could be combined with other disease parameters, such as morphological alterations, to further classify mTLE [10]. Further, the differential changes in the sub-cellular distribution of *miR-92b* in CA versus DG neurons in TLE could be explored to identify cell type-specific gene programs in TLE and to further our understanding of the gene regulatory networks that contribute toward epileptogenesis. Finally, abnormal DNA methylation signatures in neurons have been shown to drive dysregulation of gene networks in experimental and human TLE [64, 65, 141]. Accumulating evidence identifies epigenetic mechanisms as key factors through which abnormal cellular and network dysfunctions might be initiated and maintained [20]. Therefore, further dissection of the changes observed in this study (e.g., in isomiRs or snoRNAs) may not only deepen our understanding of the pathogenic mechanisms underlying the disease but could also provide novel therapeutic avenues.

## Conclusion

By exploiting human brain tissue from mTLE patients for small RNA-seq analysis, we identified previously unexplored compartment-specific changes in the expression of different classes of sncRNAs. Deregulation of these RNAs is predicted to have widespread consequences on RNA and protein expression, and therefore, our dataset constitutes a valuable resource for future studies into the role of sub-cellular changes in RNA expression in mTLE. The accumulation of *miR-92b* in the neuronal nucleolus of mTLE patient tissue triggered subsequent experiments that revealed several nucleolar changes that are indicative of nucleolar dysfunction and stress. These findings implicate nucleolar dysfunction in the pathogenesis of mTLE and identify the nucleolus as a potential therapeutic target for the treatment of epilepsy.

## Supplementary Information (we have uploaded a novel file for Supplementary file 1)

Below is the link to the electronic supplementary material.

Supplementary file1 (DOCX 126 kb)

Supplementary file2 (TIF 2086 kb)

Supplementary file3 (TIF 2618 kb)

Supplementary file4 (TIF 2737 kb)

Supplementary file5 (TIF 2795 kb)

Supplementary file6 (TIF 1992 kb)

Supplementary file7 (TIF 12861 kb)

Supplementary file8 (TIF 14451 kb)

Supplementary file9 (TIF 19296 kb)

Supplementary file10 (TIF 13282 kb)

Supplementary file11 (TIF 1556 kb)

Supplementary file12 (TIF 2088 kb)

Supplementary file13 (TIF 1838 kb)

Supplementary file14 (XLSX 10022 kb)

## Data Availability

The datasets generated for this study are deposited in the NCBI Gene Expression Omnibus (GEO) repository with reference number GSE245228 (human mTLE patient and control smallRNAseq dataset) and GSE269625 (*miR-92b* bioIP dataset).
