# Supplementary material for: Compartment-specific small non-coding RNA changes and nucleolar defects in human mesial temporal lobe epilepsy
Source: Acta Neuropathol. 2024 Nov 7;148(1):61. doi: 10.1007/s00401-024-02817-8 (PMC11543739; doi:10.1007/s00401-024-02817-8)
Supplement: Supplementary file 1 — Supplementary file1 (DOCX 126 kb) [file 401_2024_2817_MOESM1_ESM.docx]

**Supplementary information**

**Compartment-specific small non-coding RNA changes and nucleolar defects in human mesial temporal lobe epilepsy**

Vamshidhar R. Vangoor^1^, Giuliano Giuliani^1^, Marina de Wit^1^, Carolina K. Rangel^1,4^, Morten T. Venø^2,3^, Joran T Schulte^1^, Andreia Gomes-Duarte^1,5^, Ketharini Senthilkumar^1^, Noora Puhakka^1,6^, Jørgen Kjems^2^, Pierre N.E. de Graan^1^ and R. Jeroen Pasterkamp^1*^

^1^ Department of Translational Neuroscience, University Medical Center Utrecht Brain Center, University Medical Center Utrecht, Utrecht University, 3584 CG Utrecht, The Netherlands

^2^ Interdisciplinary Nanoscience Centre, Department of Molecular Biology and Genetics, 8000 Aarhus University, Denmark

^3^ Omiics ApS, 8200 Aarhus N, Denmark

^4^ Current address: Princess Máxima Center for Pediatric Oncology, Heidelberglaan 25, 3584 CS, Utrecht, the Netherlands

^5^ Current address: VectorY Therapeutics, Science Park 408, Matrix Innovation Center VI, 1098 XH, Amsterdam, The Netherlands

^6^ Current address: A.I. Virtanen Institute for Molecular Sciences, University of Eastern Finland, Kuopio, Finland

***Correspondence:**

R. Jeroen Pasterkamp: r.j.pasterkamp@umcutrecht.nl, +31-88-7568831

**Supplementary Figure Legends**

**Supplementary Fig. 1. Small RNA-seq of human TLE sub-cellular fractions.**

**a-c** Graphs showing average raw read counts, miRNA counts per sample and average number of miRNAs detected in the different samples from cytoplasm. n=6 mTLE non-HS cortex (Cx) and n=6 control Cx, n=5 mTLE non-HS hippocampus (HC) and n=5 HC control HC, and n=3 Pre Cx and n=3 Post Cx samples. Data are means +/- SD. For miRNA count data, see Supplementary excel file (Tab4).

**d-f** Graphs showing average raw read counts, miRNA counts per sample and average number of miRNAs detected in the different samples from nucleus. n=6 mTLE non-HS Cx and n=6 control Cx, n=5 mTLE non-HS HC and n=5 control HC, and n=3 Pre Cx and n=3 Post Cx samples. Data are means +/- SD. For miRNA count data, see Supplementary excel file (Tab4).

**g-h** Volcano plots showing miRNA expression changes between pre- versus postmortem-treated nuclear and cytoplasmic fractions (n=3 each), respectively.

**i-j** Volcano plots for control HC versus mTLE non-HS nucleus and cytoplasm, respectively. n=5 mTLE non-HS HC and n=5 control HC. Significantly DE genes are shown in red and blue (padj <0.05; logFC>1 and <-1). A selection of strongly upregulated (in red) or downregulated (in blue) genes are indicated in the plots.

**Supplementary Fig. 2.** **DE miRNAs in mTLE+HS samples.**

**a**-**b** Principal Component Analysis (PCA) plots for cortical (Cx) nucleus and cytoplasmic samples from controls and mTLE non-HS patients (n=6 each) based on DE miRNA expression detected by small RNA-seq.

**c-d** Volcano plots showing DE miRNAs in mTLE non-HS Cx nuclear and cytoplasmic samples. n= 6 mTLE non-HS Cx and n=5 control Cx. Significantly DE genes are shown in red and blue (padj <0.05; logFC>1 or <-1). A selection of strongly upregulated (in red) or downregulated (in blue) genes are indicated in the plots.

**e-f** PCA plots for Cx nucleus and Cx cytoplasmic samples from controls and mTLE+HS patients (n=6 each) based on DE miRNA expression detected by small RNA-seq**.**

**g-h** Heatmaps showing DE miRNAs in nuclear and cytoplasmic samples from the Cx of n=6 controls (C1, C3-7) and n=6 mTLE+HS patients (E9-14) as determined by small RNA-seq. Hierarchical clustering is shown on the top. For DE miRNAs, see Supplementary excel file (Tab8 and Tab9).

**Supplementary Fig. 3. Differential isomiR expression in mTLE hippocampus and cortex.**

**a** Heatmaps of DE miRNA isoforms (isomiRs) across the 10 hippocampal samples (n=5 controls (C1-3, C8-9) and n=5 mTLE non-HS patient (E1-5) samples) in cytoplasmic and nuclear fractions. Hierarchical clustering is shown on the top. For DE isomiRs, see Supplementary excel file (Tab12 and Tab13).

**b** Heatmap of DE miRNA isoforms across the 12 cortical samples (n=6 controls (C1, C3-7) and n=6 mTLE non-HS patient (E1-3, E6-8) samples) in nuclear fractions. For DE isomiRs, see Supplementary excel file (Tab14).

**c** Venn diagram showing the overlap of DE miRNAs between the hippocampal cytoplasmic and nuclear fractions of mTLE non-HS patients.

**d** Venn diagram showing the overlap of DE miRNAs between the hippocampal and cortical nuclear fractions of mTLE non-HS patients.

**Supplementary Fig. 4.** **Validation of differential miRNA expression in human TLE.**

**a** RT-qPCR validation of DE miRNAs detected by small RNA-seq analysis in hippocampal nuclear fractions of mTLE non-HS patients. Boxplot graphs show RT-qPCR validation and corresponding p-values of DE miRNAs. Validation was performed on samples from the same cohort (mTLE non-HS (n=5) to controls (n=5)) as used for RNA-seq as well as from additional independent samples (mTLE non-HS (n=2) to controls (n=2); n=7 each in total). Relative RNA levels were normalized to an endogenous control, *5S*, and the control group (mean value set to 1). Data are means +/- SD. Statistical differences were calculated by unpaired Welch t-test (*p < 0.05, **p < 0.01, ***p < 0.005, ****p < 0.001).

**b** RT-qPCR validation of DE miRNAs detected by small RNA-seq analysis in hippocampal cytoplasmic fractions of mTLE non-HS patients. Boxplot graphs show RT-qPCR validation, and corresponding p-values of selected DE miRNAs when comparing mTLE non-HS (n=7) to controls (n=7). Relative RNA levels were normalized to an endogenous control, *5S*, and the control group (mean value set to 1). Data are means +/- SD. Statistical differences were calculated by unpaired Welch t-test (*p < 0.05, **p < 0.01, ***p < 0.005, ****p < 0.001).

**c** Representative images showing *in situ* hybridization (ISH) for *miR-423-3p* on coronal sections of post-mortem control and mTLE non-HS hippocampus (HC). Red arrowheads indicate nuclear accumulation of *miR-423-3p* in CA neurons. White arrowheads indicate normal cytoplasmic localization in control tissue. n=1 experiment. n=2 control, n=2 mTLE non-HS. Scale bar, 50 μm.

**Supplementary Fig. 5.** **N2A cellular fractionation and biotin immunoprecipitation.**

**a** Western blot analysis to test the purity of the nuclear and cytoplasmic fractions following miRNA immunoprecipitation. Western blot on lysates of the nuclear and cytoplasmic fractions. Fibrillarin and β-tubulin were used as nuclear and cytoplasmic markers, respectively. Consistent with its subcellular localisation, fibrillarin was specifically detected in nuclear fractions, whereas the anti-β-tubulin antibody labeled the cytoplasmic fraction. n=3 experiments were performed with similar results.

**b** Barplot showing enrichment of transcripts in the nuclear and cytoplasmic fractions. *Gomafu*, *Malat1*, *Neat1,* *Gapdhint and Eef2int* were more abundant in the nuclear fraction, while *Gapdh* and *Eef2* were enriched in the cytoplasmic fraction. Enrichment is represented as a percentage of the distribution (% distribution) of a marker across the nuclear and cytoplasmic compartments. Graphs represent data from samples collected in n=3 experiments. Data are means + SD.

**c** RT-qPCR validation to test the efficiency of *miR-92b* pull-down in nuclear and total lysate samples. Boxplot graphs show a robust enrichment of *miR-92b* in cultures transfected with biotinylated *miR-92b* as compared to scrambled (Scr) control or untransfected cultures (control). Graphs represent data from samples collected in n=3 experiments. Data are means + SD.

**d** PCA showing sample clustering in nuclear and whole cell pulldown conditions (scrambled (Scr) and *miR-92b* pulldown) based on DE transcript expression detected by RNA-seq. Nuc, nuclear. n=3 samples per group.

**Supplementary Fig. 6.** **Nucleolar localization of *miR-92b.***

**a-d** Representative *in situ* hybridization (ISH) images of *miR-92b* and *miR-124-3p* in mTLE non-HS hippocampal tissue (CA1 and hilar regions). Red arrows indicate nucleolar localization of *miR-92b.* n=2 experiments with similar results. n=3 control, n=3 mTLE non-HS. Scale bar, 40 μm.

**Supplementary Fig. 7.** **Granule cell dispersion (GCD) and *miR-92b* localization in the mTLE+HS hippocampus.**

**a-c** Representative *in situ* hybridization (ISH) images for *miR-92b* in control hippocampal dentate gyrus (DG) neurons. Red boxed areas are shown at higher magnification in **d-f**.

**g-i** Representative *in situ* hybridization (ISH) images for *miR-92b* in mTLE+HS hippocampal DG neurons. Red boxed areas are shown at higher magnification in **j-l**. n=2 experiments with similar results. n=3 control, n=3 mTLE+HS samples. Scale bar, 40 μm, 20 μm (inset).

**Supplementary Fig. 8. Nucleolar localization of *miR-92b* in an experimental TLE model.**

**a** Schematic showing status epilepticus (SE) induction in the pilocarpine rat model and the different timepoints at which *in situ* hybridization (ISH) was performed (created with Biorender.com).

**b** Representative ISH images showing *miR-92b* expression in CA3 neurons in control and pilocarpine-treated rats (2-19 weeks (wk) after SE induction). Red arrows indicate nucleolar *miR-92b* signal at different timepoints. NC, negative control (scrambled-miR), devoid of signal. n=2 experiments were performed with similar results from 2 weeks (n=2 control and n=3 SE+), 4 weeks (n=2 control and n=3 SE+), 8 weeks (n=2 control and n=3 SE+) and 19 weeks (n=2 control and n=3 SE+) animals. Scale bar, 40 μm, 20 μm (inset).

**c** Representative image showing *miR-92b* nuclear localization in 2 wk SE+ pilocarpine rat tissue sections co-labelled with the nuclear marker DAPI. Red boxed area is shown at higher magnification in the lower panel. Scale bar, 40 μm, 20 μm (inset).

**d** Quantification of mean intensity ratios showing *miR-92b* expression levels in control and pilocarpine SE+ animals estimated using densitometry analysis. Mean intensities were acquired by measuring individual cell nuclear area with *miR-92b* ISH signal as a region of interest (ROI) based on corresponding DAPI area as a reference, in 12-20 cells per image from different hippocampal regions. Mean intensity ratios were calculated by normalizing the background mean intensity (BG) measured from an empty area corresponding to the size of the nuclear area for each cell and calculating the ratio over the BG using the formula: ([Mean intensity of BG ROI – Mean intensity of ROI] / Mean intensity of BG ROI). Average ratios depicted in the graph are normalized for equal area measured among all conditions. n=10 images/animal were used for the analysis from n=3 control animals from 2 wk and n=3 animals each from 2 wk, 4 wk, 8 wk and 19 wk after SE. Statistical significance was estimated by comparing mean ratios from SE+ animals of different timepoints to 2 wk controls using ANOVA and Dunnett’s T3 multiple comparisons test (*p < 0.05, **p < 0.01). ns, non-significant.

**Supplementary Fig. 9.** **Changes in nucleolar morphology in an experimental TLE model.**

**a** Representative *in situ* hybridization (ISH) images showing *miR-92b-3p* localization in the CA1 and CA3 hippocampal regions of *Tsc1-cre^-^* and *Tsc1-cre^+^* mice. n=2 experiments were performed with similar results from n=2 *Tsc1-cre^-^* and 2 *Tsc1-cre^+^* animals. Scale bar, 40 μm, 20 μm (inset).

**b** Representative images of NPM1 staining in the CA1 region of control and pilocarpine rat hippocampal tissue at 19 weeks after SE (SE+) showing changes in nucleolar size. Scale bar, 10 μm.

**c** Quantification of nucleolar size in individual CA regions (CA1, CA2 and CA3) at 2 weeks after SE. n=26, n=18, n=23 (CA1, CA2, CA3) images from n=4 control rats, and n=22, n=10, n=15 (CA1, CA2, CA3) images from n=4 SE+ rats. Data are means +/- SD. ****P<0.0001. ns, not significant. Multiple Mann-Whitney U test.

**d** Quantification of nucleolar size in individual CA regions (CA1, CA2 and CA3) at 19 weeks after SE. n=15, n=9, n=13 (CA1, CA2, CA3) images in n=2 control rats, and n=33, n=14, n=20 (CA1, CA2, CA3) images from n=3 SE+ rats. Data are means +/- SD. ****P<0.0001. ***p<0.001. ns, not significant. Multiple Mann-Whitney U test.

**e** Quantification of NPM1 mean intensity estimated between controls and mTLE tissue. Each dot represents a mean value estimated from an individual images (control n=23 images, mTLE non-HS n=23 images, mTLE+HS n=25 images with at least 5 images per individual) collected from n=4 controls, and n=4 patient samples/group. Data are means +/- SD. ns, not significant. Ordinary one-way ANOVA with Sidak’s multiple comparisons test.

**Supplementary Fig. 10. Nucleolar characterization using C23 immunostaining.**

**a-b** Boxplots showing the quantification of C23 immunofluorescence in sub-cellular compartments of the CA3 and CA4 regions. Increased C23 signal in the soma and a reduced signal in the nucleolus is observed in TLE compared to Control. Fluorescence signal in the nucleus was unchanged, indicating a redistribution of the C23 in TLE tissue. ANOVA with Sidaks multiple comparisons test. Data are means +/- SD. ns, not significant, ****p<0.0001. n=54 cells were measured from n=4 control and n=3 mTLE non-HS tissues.

**c** Boxplots showing the quantification of the size of the nucleolus based on C23 immunofluorescence. Increased nucleoli size is observed in the CA1, CA3 and CA4 regions of the mTLE non-HS hippocampus. ANOVA with Sidaks multiple comparisons. Data are means +/- SD. ****p<0.0001. n (cells measured) =54 from control and mTLE non-HS (CA1), n=26 and n=36 in control and mTLE non-HS samples, respectively (CA3), and n=28 and n=29 from control and mTLE non-HS samples, respectively, (CA4) from n=4 controls and n=3 mTLE non-HS tissues.

**d** Nuclear size is unaltered in regions of the hippocampus in which an increase in nucleolar size was observed. n=4 controls and n=3 mTLE non-HS tissues. ANOVA with Sidaks multiple comparisons test. Data are means +/- SD. ns, not significant.

**e** Representative Western blots probed with antibodies against CBX4, CBX8 and β-actin on total lysates from control and mTLE non-HS hippocampus.

**f-g** Quantification of CBX4 and CBX8 protein levels comparing the different patient groups with controls. n=6 controls and n=6 mTLE non-HS samples. Data are means +/- SD. ns, not significant. ANOVA with Kruskal-Wallis test.

**Supplementary Fig. 11. ncRNA analysis from the small RNA-seq data.**

**a** Schematic overview of the workflow of small RNAseq analysis enriching for non-coding (nc) RNAs and SnoRNAs.

**b** Percentage of assigned reads for ncRNA from controls (n=4) and mTLE non-HS patients (n=5). Control cyt = control cytoplasm, Control nuc = control nucleus, mTLE non-HS cyt = mTLE non-HS cytoplasm, mTLE non-HS nuc = mTLE non-HS nucleus. Data are means +/- SD.

**c** Dispersion plot for nuclear and cytoplasmic fractions based on DE small ncRNAs detected by small RNA-seq. The black dots represent gene-wise estimates of dispersions. The fitted values for mean of the counts and mean of dispersion are shown by the red dots. The black points are then shrunken to fit within the red line and these values are used for the next step, the statistical test for DE genes (Wald test).

**d** plotMA for nuclear and cytoplasmic fractions based on DE ncRNAs detected by small RNA-seq. Dots marked in blue represent genes with padj<0.1.

**Supplementary Fig. 12. Differential expression of SnoRNAs in human mTLE non-HS cytoplasmic fractions.**

**a** Differentially expressed (DE) SnoRNAs in the cytoplasmic fraction of TLE hippocampal (HC) samples. Heatmap showing differentially expressed SnoRNAs in cytoplasmic fractions from control and mTLE non-HS HC samples. Controls (n=4; C1-C3, C9) and mTLE non-HS patients (n=5; E1-E5). For DE snoRNA list, see Supplementary excel file (Tab19).

**b** Normalized transcripts per million (TPM) counts of the three common DE SnoRNAs in the cytoplasmic fraction. n=4 controls and n=5 mTLE non-HS samples. Data are means +/- SD. Unpaired t-test. p-values are depicted in each graph.

**c** *Npm1* knockdown (KD) using the Crispr-Cas13 system leads to deregulation of SnoRNA expression. Graph showing the relative expression of *Npm1* in N2A cells transfected with different guide RNAs (G1-G3). Data are normalized to the house-keeping gene *Hprt1*. Transfection with G2 results in a significant KD of *Npm1*, in comparison with a control transfection condition (NS). n=10 transfections for NS and n=11 transfections per guide from n=4 experiments. Data are shown as pooled from different experiments. Data are means +/- SD. Unpaired t-test, **p<0.01.

**d** Representative Western Blot for NPM1 and fibrillarin on lysates from N2A cells transfected with NS and G2 guides. β-actin was used as loading control. Two different experiments are shown.

**e** Quantification of Western blots as in **d** showing normalized protein levels (to β-actin) for NPM1 after NS and G2 transfection. n=7 transfections per condition (NS and G2) from 2 experiments. Data are means +/- SD. Unpaired -test, **p<0.01.

**f-h** Relative expression of SnoRNAs after *Npm1* KD. Data are normalized to the housekeeping gene *Hprt1*. n=10 transfections per condition (NS and G2) from 4 independent experiments. Data are means +/- SD. Unpaired t-test, *p<0.05.

**Supplementary Methods**

**Pilocarpine rat model**

The pilocarpine rat model was generated as part of previous studies [4, 5]. Paraffin preserved tissue sections were used for studying *miR-92b-3p* expression by *in situ* hybridization and for assessing nucleolar morphology with NPM1 immunostaining. Briefly, status epilepticus (SE) was induced in 21 days old male Wistar rats (Charles River Laboratories) by intraperitoneal (i.p.) administration of lithium chloride (Li^+^) 18-20 h before subcutaneous injection of pilocarpine (40 mg/kg, Merck). Methyl-scopolamine (1 mg/kg, Sigma-Aldrich) was injected 30 minutes before the injection of pilocarpine to reduce the peripheral effects of pilocarpine. Subsequently the behavior of pilocarpine injected rats was classified according to the six stages of the Racine scale [11]. Finally, to suppress seizures and to reduce mortality, rats received an injection of diazepam (4 mg/kg, i. p., Centrafarm Services BV) after 1 h of SE. Only rats that reached stage 6 of seizures according to the Racine scale were considered as SE+ animals. Rats were monitored by video recording and from 15-18 weeks onwards pilocarpine injected rats displayed spontaneous recurrent seizures (SRS). Rats were also monitored for handling-induced seizures several times a day. In 44% of SE+ animals, SRS were detected. Control animals were injected with an equal volume of saline instead of pilocarpine. Experimental procedures were approved by local authorities and in line with European regulation. In the current study, paraffin-embedded tissue sections from different time points after SE induction (2, 4, 8 and 19 weeks) and their corresponding control sections were used for analysis.

***Tuberous sclerosis complex 1* (*Tsc1*) knockout mice**

Tissue sections from *Tuberous sclerosis complex 1* (Tsc1) knockout mice were used that lack expression of *Tsc1* in neurons. *Tsc1* knockout mice (*Tsc1*-ko) were generated as part of previous studies [7]. Briefly, inducible *Tsc1* knockout mice were obtained by crossing conditional biallelic floxed *Tsc1* mutant mice (Tsc1^tm1Djk^; MGI:2656240) [2] with tamoxifen inducible Cre^ERT2^ mice under the neuronal *CamK2a* promotor (Tg(Camk2a-cre/ERT2)2Gsc; MGI:3759305) [10] in a C57bl/6j background. Resulting mice were *Tsc1*^f/f^-Tg (*Camk2a-Cre^ERT2+^*) or *Tsc1^f/f^-Tg* (*Camk2a-Cre^ERT2-^*), named *Tsc1-Cre^+^* and *Tsc1-Cre^-^*, respectively. Cre recombinase was absent in *Tsc1-Cre^-^* animals and these were used as controls. Deletion of *Tsc1* gene was induced in 12-20 week old mice. Seizures were observed starting between day 8 and day 12 after gene deletion. Tonic-clonic seizures, characterized by generalized limb clonus and loss of posture, were observed in *Tsc1-Cre^+^* mice. All *Tsc1* knockout animals died between day 12 and 18 immediately after a tonic-clonic seizure, characterized by immobility and an abrupt flattening of electroencephalogram (EEG) activity [7]. Mice were perfused transcardially at day 12 after gene deletion with 4% paraformaldehyde in 1x PBS. Brains were isolated, incubated in 10% sucrose overnight and embedded in a solution with 12% gelatin and 10% sucrose. Experimental procedures performed on mouse brain tissue were approved by local authorities, in accordance with the institutional guidelines (license# AVD1010020172684) at the Erasmus MC university medical center, Rotterdam, The Netherlands. 20 µm thick brain sections were obtained on a cryostat (Catalogue# CM 1950, Leica Biosystems) and collected on glass slides and stored at -80^o^C until use. ISH for *miR-92b* was performed as described in the Methods section of the manuscript.

**Design and generation of plasmids with guide RNAs for NPM1 knockdown**

For transient knockdown of NPM1 in murine cell lines, we exploited the CRISPR-Cas13 system [1]. Guide RNA design and cloning was performed as described previously [3]. Briefly, guides were designed by using Cas13Design (https://cas13design.nygenome.org/) and CHOPCHOP (https://chopchop.cbu.uib.no/) [9, 14], based on the *NPM1* reference sequence for mouse *NPM1* (NM_001252260.1). Guides for mouse NPM1 were selected based on the longest available transcript (*ENSMUST00000075641.9*). Next we applied the following criteria for selecting guides that will result in good targeting efficiency: 1) guides with higher score value, 2) minimal off-target score, and 3) hybridizing to coding regions. Three guide sequences targeting *NPM1* were selected based on these criteria. Sequences of the different guides with 5’ sequence overhangs AAAC (forward primer) and AAAA (reverse primer) nucleotides, required for ligation into the CasRx plasmid, are provided in Supplementary Table 5.

For the generation of guide RNA plasmids, forward and reverse guide oligonucleotides (2 mg each, IDT DNA) were annealed using 1x annealing buffer (100 mM Tris, pH 7.5-8.0, 1 M NaCl, 10 mM EDTA) pH 7.5 and water in a final volume of 50 µl. Annealing was performed at 95^o^C for 5 min and samples were left to cool down to RT (approximately 2.5 h). The vector used for guide RNA cloning, *p-U6BbsI-CasRx*, was generated as described previously [3]. Ten µg of *p-U6BbsI-CasRx* was restriction digested with *BbsI* (Catalogue# R0539S, NEB) overnight at 37^o^C in reaction buffer. Then the reaction was run in 2% agarose gel, excised and purified using Purelink^TM^ (Catalogue#K210012, ThermoFischer Scientific) according to manufacturer’s protocol. For ligation, 1 µl of annealed oligos (1 in 5 diluted) were ligated in a 1 to 10 plasmid:insert ratio with *p-U6BbsI-CasRx* plasmid using the T4 DNA ligase kit (Catalogue#18005025, ThermoFisher Scientific) for 1 h at RT. Finally, bacterial transformation and sequence verification with Sanger sequencing (Macrogen Europe B.V.) were performed.

**Cell culture and cell lines**

Mouse Neuro2A neuroblastoma cells (RRID:CVCL_0470) were purchased from ATCC (CCL-131^™^). Cells were cultured in Dulbecco's modified Eagle's medium (DMEM), containing 10% FBS (ThermoFisher Scientific) and penicillin/streptomycin (100 U/ml and 100 mg/ml, respectively) at 37^o^C and 5% CO2. For Guide RNA transfections, Neuro2A cells (1.5 x 10^5^ cells/ml) were plated and transfected after 24 h. 250 ng plasmid expressing guide RNAs and 250 ng pXR001 *EF1a-CasRx-2A-EGFP* (pXR001: EF1a-CasRx-2A-EGFP was a gift from Patrick Hsu (Addgene plasmid # 109049; http://n2t.net/addgene:109049; RRID:Addgene_109049 [8]) expressing CRISPR-Cas13, were co-transfected using the Lipofectamine 2000 transfection reagent (ThermoFisher Scientific) according to manufacturer’s instructions. After 48 h, cells were harvested for gene expression analysis in 300 μl Qiazol and stored at -80^o^C until RNA extraction. RNA extraction, RT-qPCR and estimation of NPM1 KD were performed as described in the Methods section of the manuscript. Similarly, levels of selected SnoRNAs (*snord14e*, *snord35b*, *snord49a*) after NPM1 KD were estimated by RT-qPCR as described. Primer sequences for *NPM1*, *SnoRNAs* and housekeeping genes are provided in Supplementary Table 1. For quantification of protein levels three wells treated with the same transfection mix were pooled. Cells were lysed in RIPA buffer by scraping and by passing through a 27 g needle (BD Plastipak). The supernatant was collected by centrifugation at 13.200 g for 15 min at 4ºC. Western blot analysis for NPM1 and β-actin was performed as described in the Methods section of the manuscript.

**Supplementary Table 1:** RT-qPCR primers used in the study.

| **Primer name** | **Sequence (5'-3')** |
| --- | --- |
| h*GAPDH*_intr_fw | GGGCCTCACTCCTTTTGC |
| h*GAPDH*_fw | TGGAAGGACTCATGACCACA |
| h*GAPDH*_rev | GGGATGATGTTCTGGAGAGC |
| h*GOMAFU*_fw | GTGTGTGTCTGCTGAGGTG |
| h*GOMAFU*_rev | CTGGGGTTAGTAAGAAGAGAA |
| h*NEAT1*_fw | TCGGGTATGCTGTTGTGAAA |
| h*NEAT1*_rev | TGACGTAACAGAATTAGTTCTTACCA |
| h*MALAT1*_fw | GACGGAGGTTGAGATGAAGC |
| h*MALAT1*_rev | ATTCGGGGCTCTGTAGTCCT |
| m*Gapdh*_fw | CGTAGACAAAATGGTGAAGG |
| m*Gapdh*_rev | AGTGGAGTCATACTGGAACA |
| m*Gapdh*_intr_fw | TACAGACCCATGAGGAGTTC |
| m*Gapdh*_intr_rev | GTAGACCATGTAGTTGAGGTC |
| m*Neat1_*fw | CACACGCTTCTCTGTACTAA |
| m*Neat1*_rev | TGACAGAGGTCGAGAATGTA |
| m*Malat1*_fw | CCTAATCACAGACCCTTCAC |
| m*Malat1*_rev | AAATTACAGGCAAGGGGAAA |
| m*Eef2*_fw | TGCATCATTGAGGAGTCTGG |
| m*Eef2*_rev | CGATATGACACAACAGGGT |
| m*Eef2*_intr_fw | CTCATCCTTCTGCCTATGTG |
| m*Eef2*_intr_rev | ATATGACACAACAGGGTCAG |
| m*Npm1*_fw | TTCCTTGGCGTGATTCCGTC |
| m*Npm1*_rev | GCTCATTTTCATCATTATCCACGAA |
| m*Hprt1*_fw | ATGGGAGGCCATCACATTGT |
| m*Hprt1*_rev | ATGTAATCCAGCAGGTCAGCAA |
| m*Snord14e*_fw | CGGTTTCCACCAGAACGCAA |
| m*Snord14e*_rev | CATCCAAGGAAGGTAACTGC |
| m*Snord35b*_fw | TCTGTTCTCACGATGGTCTTCA |
| m*Snord35b*_rev | GCATCAGCTTTGTCAACTGGC |
| m*Snord38a*_fw | CTGTCCAGTTCTGCTACTG |
| m*Snord38a*_rev | CAGCACTAAAGTGTTCATC |
|  |  |
| h - human, m - mouse |  |

**Supplementary Table 2:** ncRNA and snoRNA mapping rates according to different k-mers sizes.

|  | **ncRNA** | | | | | **snoRNAs** | | |
| --- | --- | --- | --- | --- | --- | --- | --- | --- |
|  | K-mer size | | | | | | | |
| **Sample ID** | 13 | 15 | 17 | 19 | 23 | 13 | 15 | 19 |
| C3 cyt | 70% | 57% | 56% | 52% | 21% | 0,54% | 0,52% | 0,47% |
| C3 nuc | 79% | 73% | 72% | 70% | 24% | 9,15% | 9,09% | 8,66% |
| C1 cyt | 70% | 58% | 57% | 53% | 25% | 0,71% | 0,67% | 0,60% |
| C1 nuc | 81% | 75% | 74% | 72% | 23% | 8,34% | 8,29% | 7,90% |
| C2 cyt | 69% | 59% | 58% | 54% | 26% | 0,65% | 0,70% | 0,62% |
| C2 nuc | 83% | 78% | 77% | 75% | 23% | 10,38% | 10,31% | 9,75% |
| E3 cyt | 62% | 52% | 51% | 48% | 19% | 1,02% | 0,97% | 0,85% |
| E3 nuc | 63% | 56% | 56% | 53% | 22% | 10,81% | 10,69% | 10,11% |
| E2 cyt | 65% | 52% | 51% | 47% | 22% | 0,76% | 0,73% | 0,64% |
| E2 nuc | 69% | 64% | 63% | 60% | 21% | 9,86% | 9,73% | 9,70% |
| E1 cyt | 56% | 44% | 43% | 39% | 22% | 1,02% | 0,97% | 0,86% |
| E1 nuc | 69,5 | 66% | 65% | 63% | 22% | 13,04% | 12,92% | 12,08% |

**Supplementary Table 3:** Representative top 10 gene TPM (transcripts per million) average counts per k-mer (percentage relative to k-mer 13) in n= nuclear and n=3 cytoplasmic fractions from control and mTLE non-HS samples.

|  | K-mer 13 | K-mer 15 | K-mer 19 |
| --- | --- | --- | --- |
| C3 nuc | 100,00 | 100,13 | 98,43 |
| C1 nuc | 100,00 | 100,14 | 98,63 |
| C2 cyt | 100,00 | 100,83 | 105,00 |
| C2 nuc | 100,00 | 100,31 | 96,48 |
| E3 nuc | 100,00 | 100,17 | 97,63 |
| E3 cyt | 100,00 | 100,00 | 105,61 |
| E2 nuc | 100,00 | 100,16 | 97,65 |
| E1 cyt | 100,00 | 100,66 | 107,24 |
| E1 nuc | 100,00 | 100,11 | 101,40 |

**Supplementary Table 4:** Potential methylation targets of DE SnoRNAs predicted by Plexy [6].

| SnoRNA | Gene | Plexy  (Interaction energy) | TLE  (LogFold_2_  Change) | Gene function |
| --- | --- | --- | --- | --- |
| *snoRD14E* | *KCNT1* | -21.5 | no | Sodium-activated potassium channel subunit [12, 13] |
|  | *RNA18S* | no | no | rRNA subunit |
|  | *RNA28S* | no | no | rRNA subunit |
|  | *XIRP1* | -20.3 | +4.71 | Protects actin filaments during depolymerization [12, 13] |
| *snoRD35B* | *AGAP3* | -18.1 | no | Essential component of the N-methyl-D-aspartate (NMDA) receptor signaling complex [12, 13] |
|  | *TARS3* | -22.7 | no | Enables threonine-tRNA ligase activity [12, 13] |
|  | *RNA28S* | no | no | rRNA subunit |
|  | *CCL2* | -18.5 | +3.59 | Ligand for C-C chemokine receptor [12, 13] |
| *snoRD38A* | *RNA5S* | -18.7 | -5 | rRNA subunit |
|  | *KCNT1* | -20,1 | no | Sodium-activated potassium channel subunit [12, 13] |
|  | *RNA28S* | no | no | rRNA subunit |
|  | *NPAS4* | -22.2 | +3.81 | Transcription factor that regulates the excitatory-inhibitory balance within neural circuits [12, 13] |

**Supplementary Table 5: Guide RNA sequences for targeting *Npm1* mRNA.**

| **Guide** | **Oligo name** | **Sequence (5'-3')** |
| --- | --- | --- |
| mG1 | gRNA_m*Npm1*_fw1 | AAACTTGCATTATAAAAAGGACAGCCA |
|  | gRNA_m*Npm1*_rv1 | AAAATGGCTGTCCTTTTTATAATGCAA |
| mG2 | gRNA_m*Npm1*_fw2 | AAACACAGACATTTTCAAAGTTGCCAG |
|  | gRNA_m*Npm1*_rv2 | AAAACTGGCAACTTTGAAAATGTCTGT |
| mG3 | gRNA_m*Npm1*_fw3 | AAACGACGGAATCACGCCAAGGAA |
|  | gRNA_m*Npm1*_rv3 | AAAATTCCTTGGCGTGATTCCGTC |

**m - mouse, G - guide.**

**Supplementary References**

1. Abudayyeh OO, Gootenberg JS, Essletzbichler P, Han S, Joung J, Belanto JJ, Verdine V, Cox DBT, Kellner MJ, Regev A, Lander ES, Voytas DF, Ting AY, Zhang F (2017) RNA targeting with CRISPR–Cas13. Nature 550:280–284. doi: 10.1038/nature24049

2. Erdmann G, Schütz G, Berger S (2007) Inducible gene inactivation in neurons of the adult mouse forebrain. BMC Neurosci 8:63. doi: 10.1186/1471-2202-8-63

3. Gomes-Duarte A, Venø MT, de Wit M, Senthilkumar K, Broekhoven MH, van den Herik J, Heeres FR, van Rossum D, Rybiczka-Tesulov M, Legnini I, van Rijen PC, van Eijsden P, Gosselaar PH, Rajewsky N, Kjems J, Vangoor VR, Pasterkamp RJ (2022) Expression of Circ_Satb1 Is Decreased in Mesial Temporal Lobe Epilepsy and Regulates Dendritic Spine Morphology. Front Mol Neurosci 15:1–21. doi: 10.3389/fnmol.2022.832133

4. van der Hel WS, Hessel EVS, Bos IWM, Mulder S, Verlinde SAMW, van Eijsden P, de Graan PNE (2014) Persistent reduction of hippocampal glutamine synthetase expression after status epilepticus in immature rats. European Journal of Neuroscience 40:3711–3719. doi: 10.1111/ejn.12756

5. Kan AA, van der Hel WS, Kolk SM, Bos IWM, Verlinde SAMW, van Nieuwenhuizen O, de Graan PNE (2012) Prolonged increase in rat hippocampal chemokine signalling after status epilepticus. J Neuroimmunol 245:15–22. doi: 10.1016/J.JNEUROIM.2012.01.012

6. Kehr S, Bartschat S, Stadler PF, Tafer H (2011) PLEXY: efficient target prediction for box C/D snoRNAs. Bioinformatics 27:279–280. doi: 10.1093/BIOINFORMATICS/BTQ642

7. Koene LMC, van Grondelle SE, Proietti Onori M, Wallaard I, Kooijman NHRM, van Oort A, Schreiber J, Elgersma Y (2019) Effects of antiepileptic drugs in a new TSC/mTOR-dependent epilepsy mouse model. Ann Clin Transl Neurol 6:1273–1291. doi: 10.1002/acn3.50829

8. Konermann S, Lotfy P, Brideau NJ, Oki J, Shokhirev MN, Hsu PD (2018) Transcriptome Engineering with RNA-Targeting Type VI-D CRISPR Effectors. Cell 173:665-676.e14. doi: 10.1016/J.CELL.2018.02.033

9. Labun K, Montague TG, Krause M, Torres Cleuren YN, Tjeldnes H, Valen E (2019) CHOPCHOP v3: expanding the CRISPR web toolbox beyond genome editing. Nucleic Acids Res 47:W171–W174. doi: 10.1093/nar/gkz365

10. Meikle L, Talos DM, Onda H, Pollizzi K, Rotenberg A, Sahin M, Jensen FE, Kwiatkowski DJ (2007) A Mouse Model of Tuberous Sclerosis: Neuronal Loss of Tsc1 Causes Dysplastic and Ectopic Neurons, Reduced Myelination, Seizure Activity, and Limited Survival. The Journal of Neuroscience 27:5546–5558. doi: 10.1523/JNEUROSCI.5540-06.2007

11. Racine RJ (1972) Modification of seizure activity by electrical modification of after-discharge. Electroencephalogr Clin Neurophysiol 32:281–294

12. Safran M, Rosen N, Twik M, BarShir R, Stein TI, Dahary D, Fishilevich S, Lancet D (2022) The GeneCards Suite. Practical Guide to Life Science Databases 27–56. doi: 10.1007/978-981-16-5812-9_2/FIGURES/8

13. Stelzer G, Rosen N, Plaschkes I, Zimmerman S, Twik M, Fishilevich S, Iny Stein T, Nudel R, Lieder I, Mazor Y, Kaplan S, Dahary D, Warshawsky D, Guan-Golan Y, Kohn A, Rappaport N, Safran M, Lancet D (2016) The GeneCards Suite: From Gene Data Mining to Disease Genome Sequence Analyses. Curr Protoc Bioinformatics 54:1.30.1-1.30.33. doi: 10.1002/CPBI.5

14. Wessels HH, Méndez-Mancilla A, Guo X, Legut M, Daniloski Z, Sanjana NE (2020) Massively parallel Cas13 screens reveal principles for guide RNA design. Nat Biotechnol 38:722–727. doi: 10.1038/s41587-020-0456-9
