# Supplementary figures and images for: Compartment-specific small non-coding RNA changes and nucleolar defects in human mesial temporal lobe epilepsy
Source: Acta Neuropathol. 2024 Nov 7;148(1):61. doi: 10.1007/s00401-024-02817-8 (PMC11543739; doi:10.1007/s00401-024-02817-8)

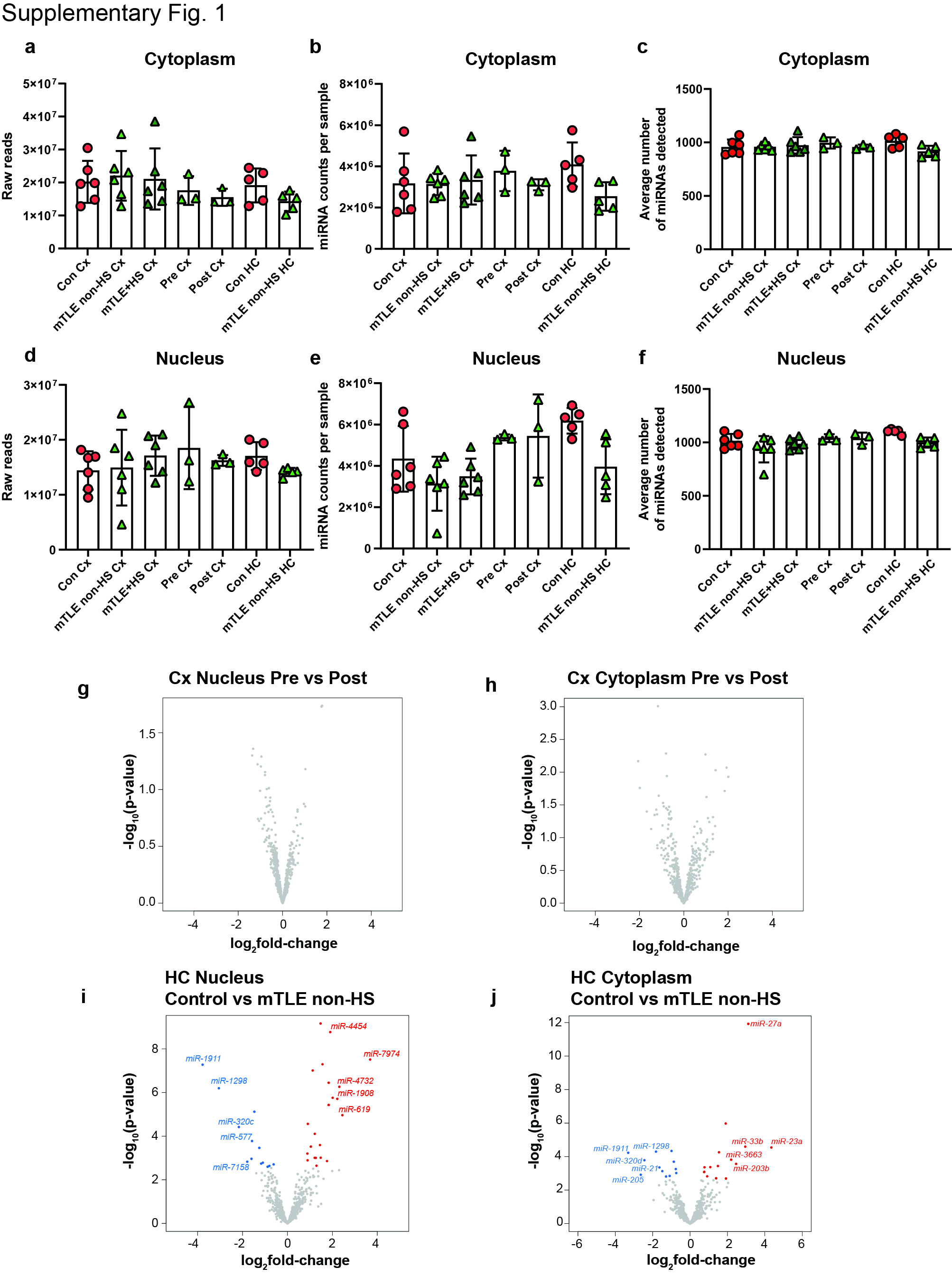

Supplement: Supplementary file 2 — Supplementary file2 (TIF 2086 kb) [file 401_2024_2817_MOESM2_ESM.tif]

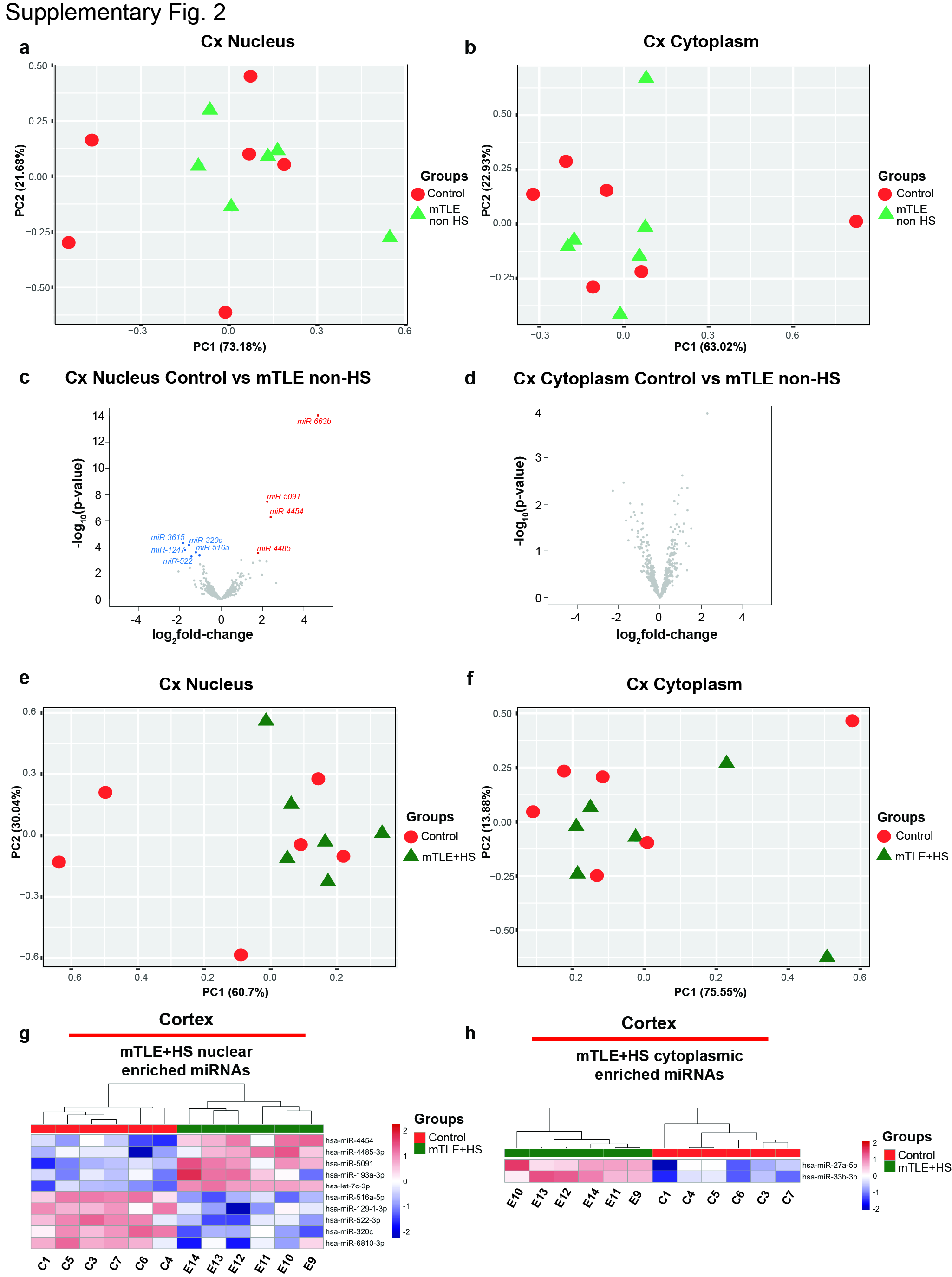

Supplement: Supplementary file 3 — Supplementary file3 (TIF 2618 kb) [file 401_2024_2817_MOESM3_ESM.tif]

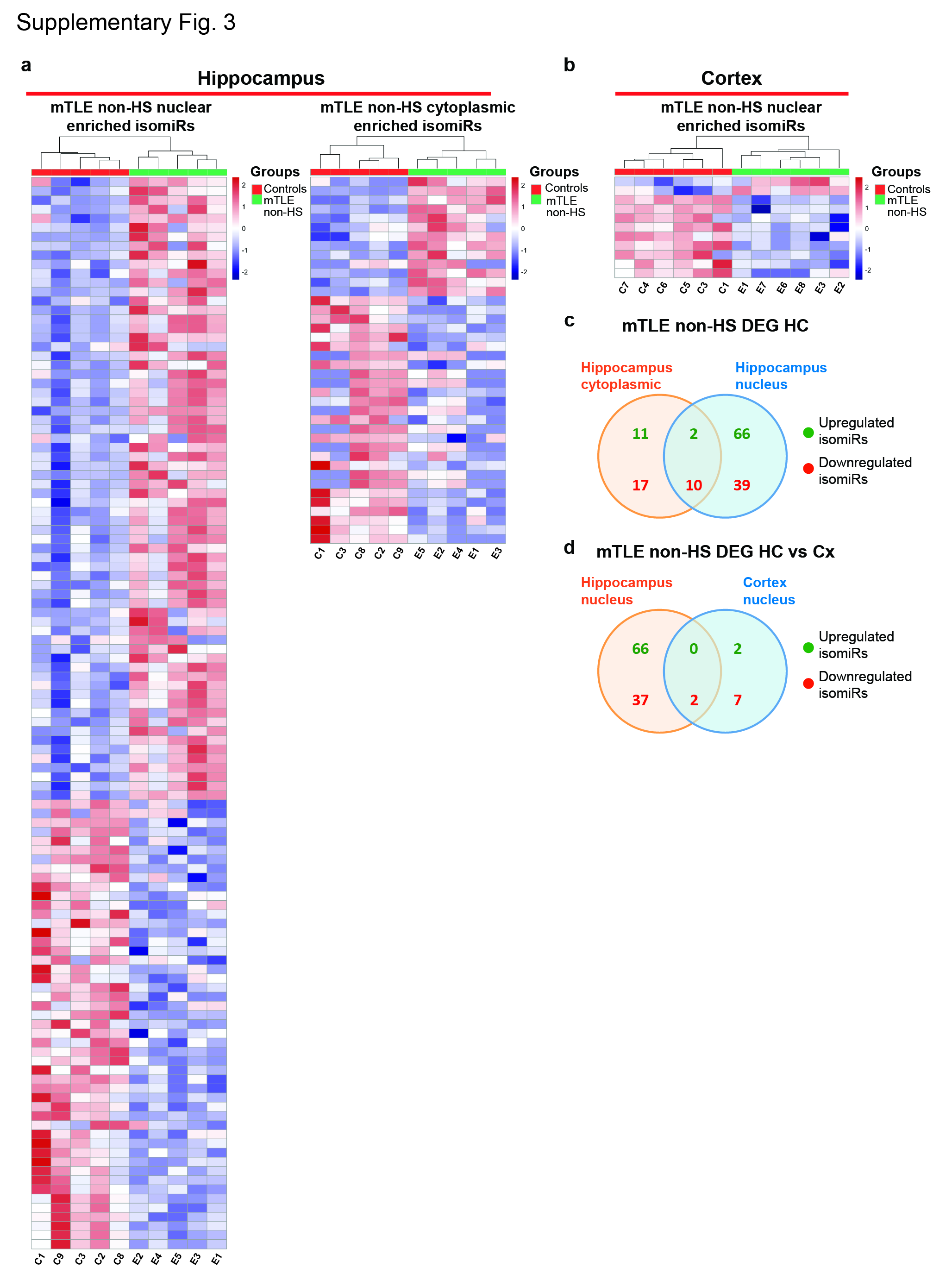

Supplement: Supplementary file 4 — Supplementary file4 (TIF 2737 kb) [file 401_2024_2817_MOESM4_ESM.tif]

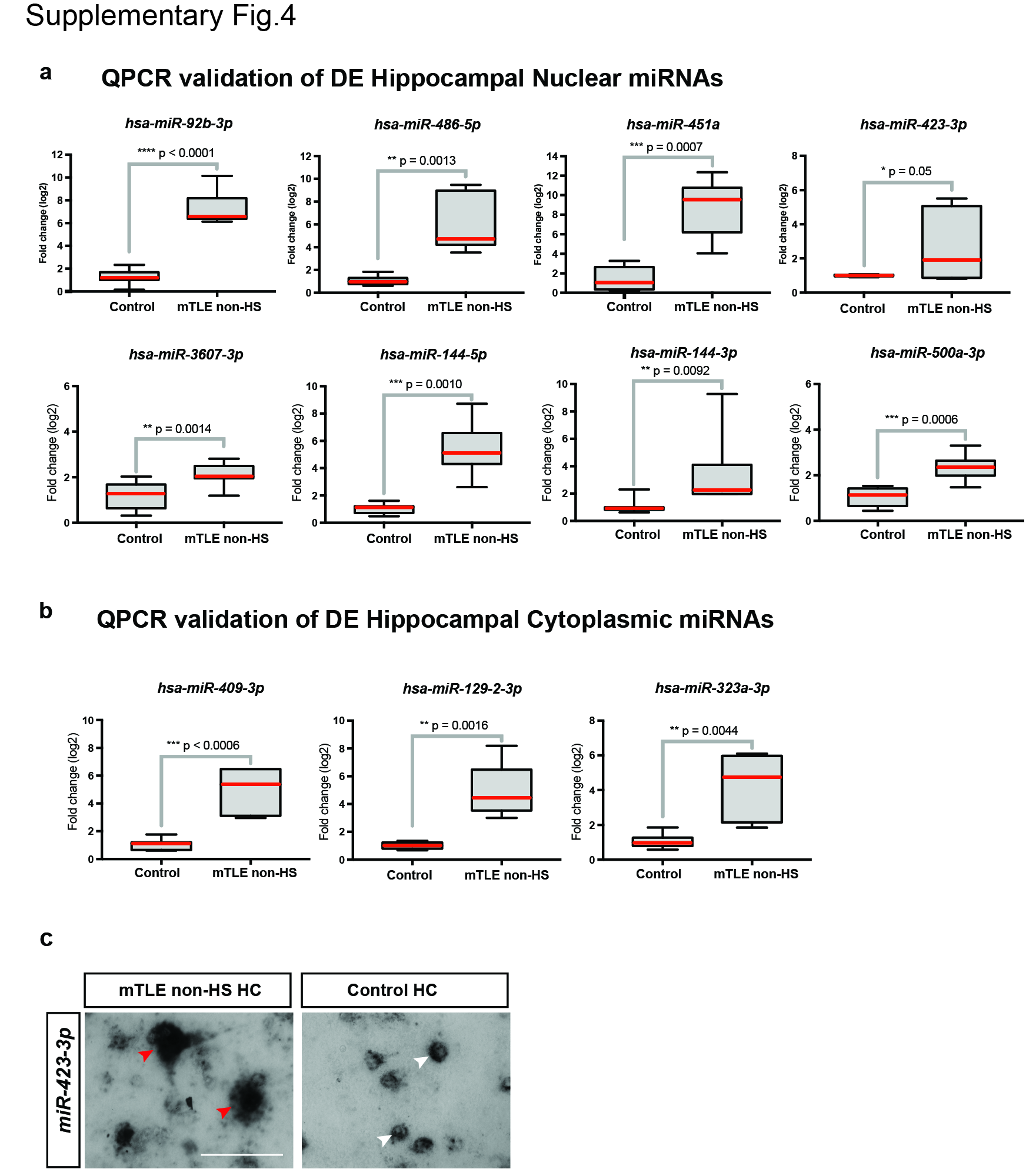

Supplement: Supplementary file 5 — Supplementary file5 (TIF 2795 kb) [file 401_2024_2817_MOESM5_ESM.tif]

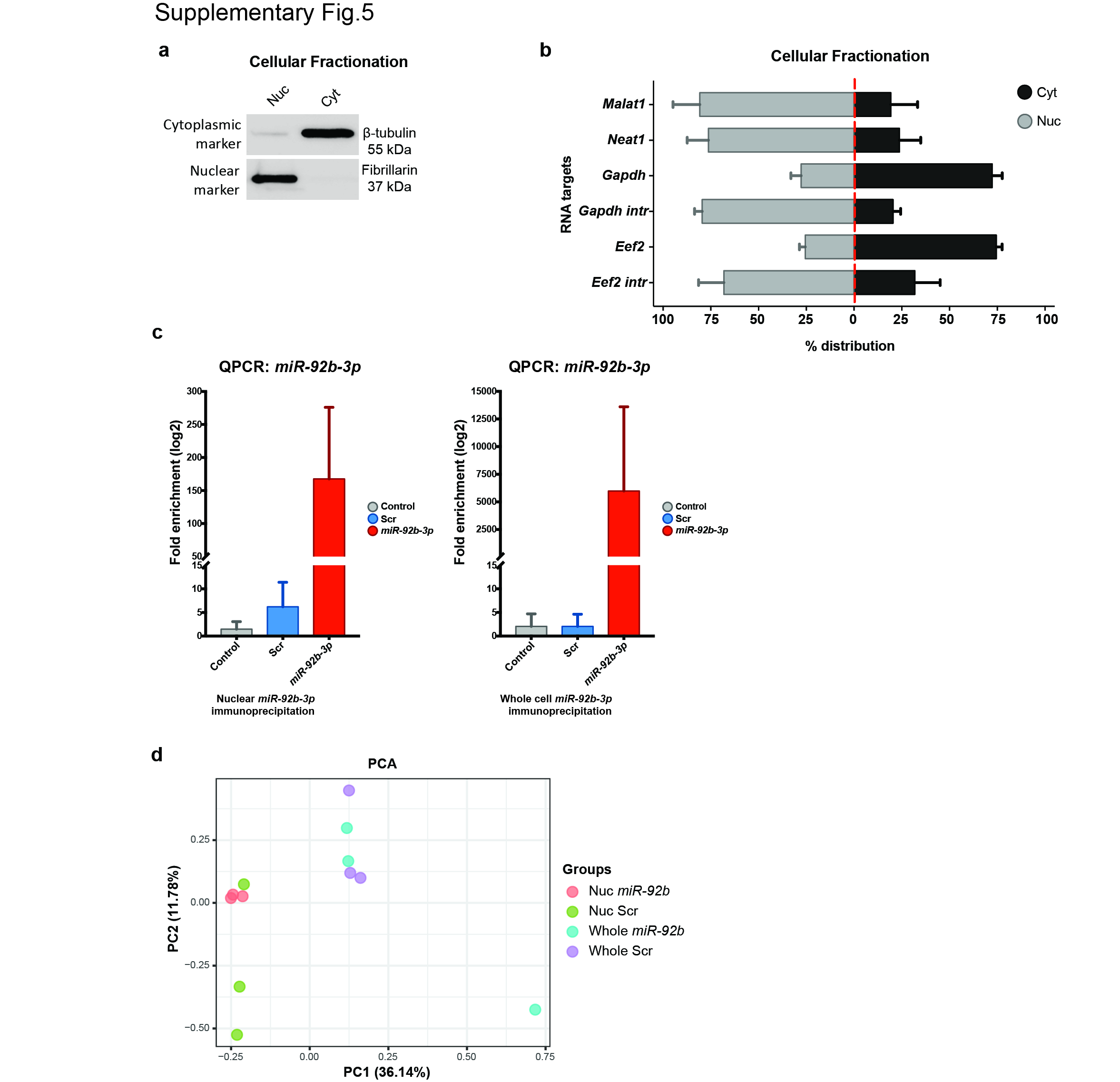

Supplement: Supplementary file 6 — Supplementary file6 (TIF 1992 kb) [file 401_2024_2817_MOESM6_ESM.tif]

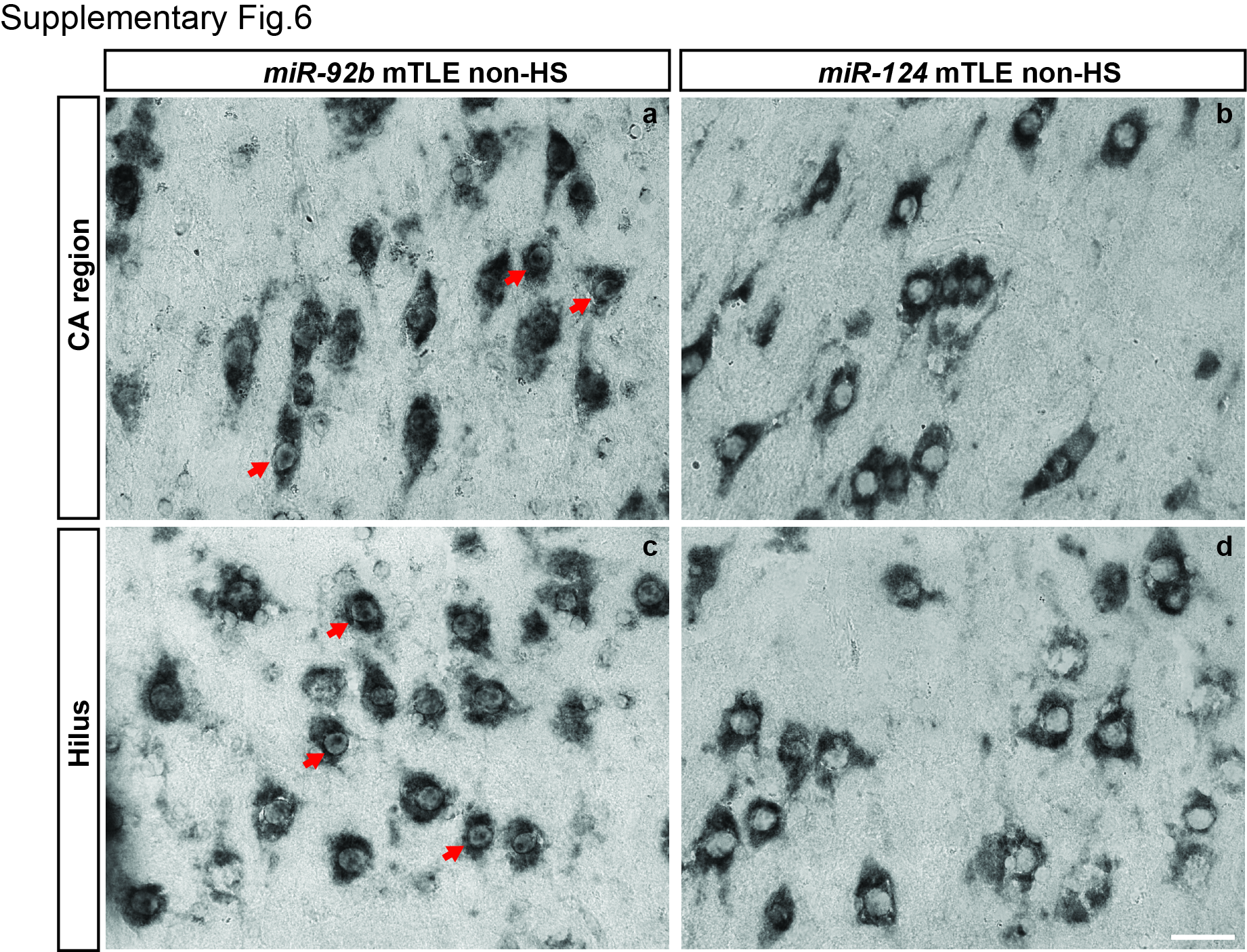

Supplement: Supplementary file 7 — Supplementary file7 (TIF 12861 kb) [file 401_2024_2817_MOESM7_ESM.tif]

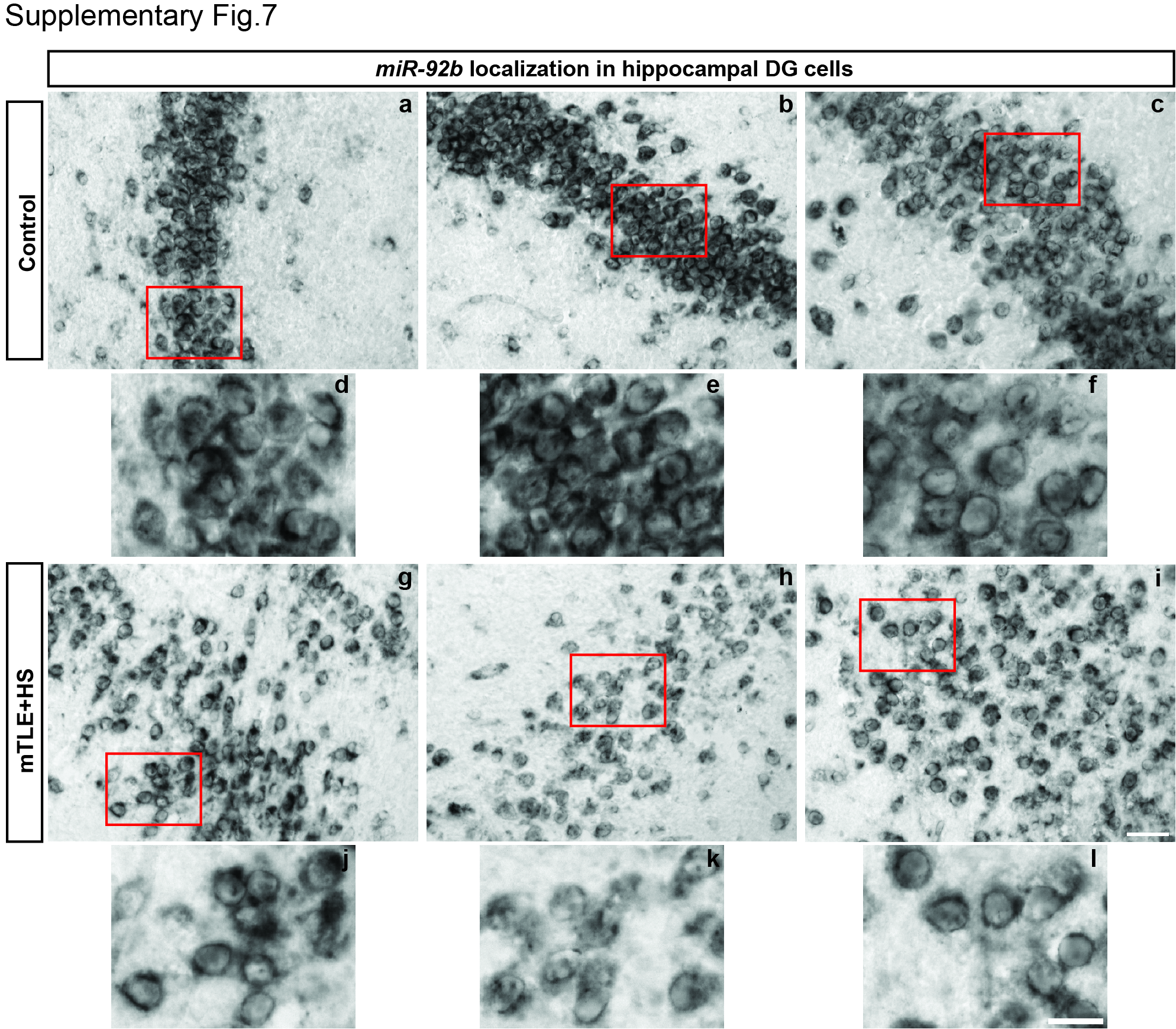

Supplement: Supplementary file 8 — Supplementary file8 (TIF 14451 kb) [file 401_2024_2817_MOESM8_ESM.tif]

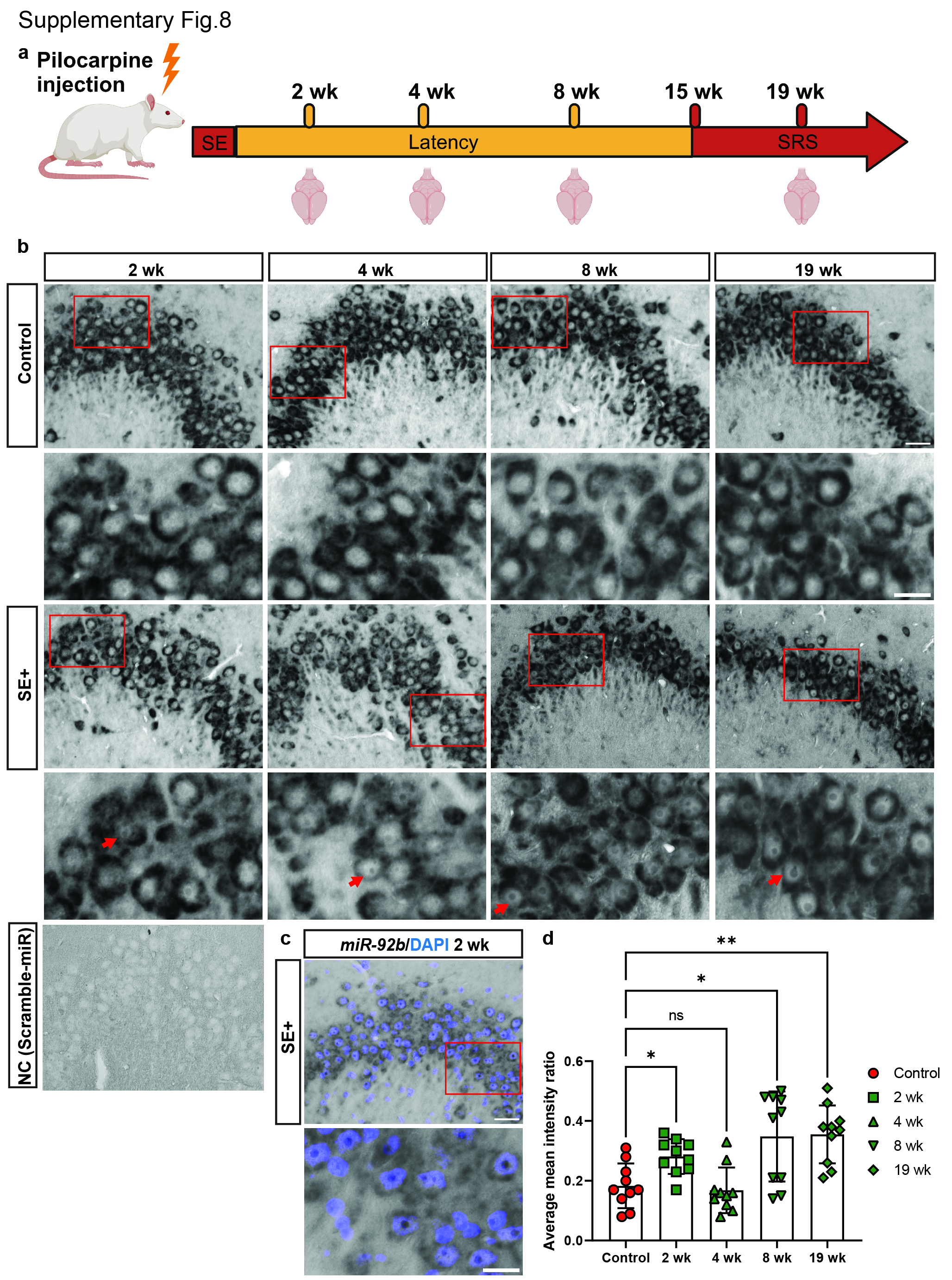

Supplement: Supplementary file 9 — Supplementary file9 (TIF 19296 kb) [file 401_2024_2817_MOESM9_ESM.tif]

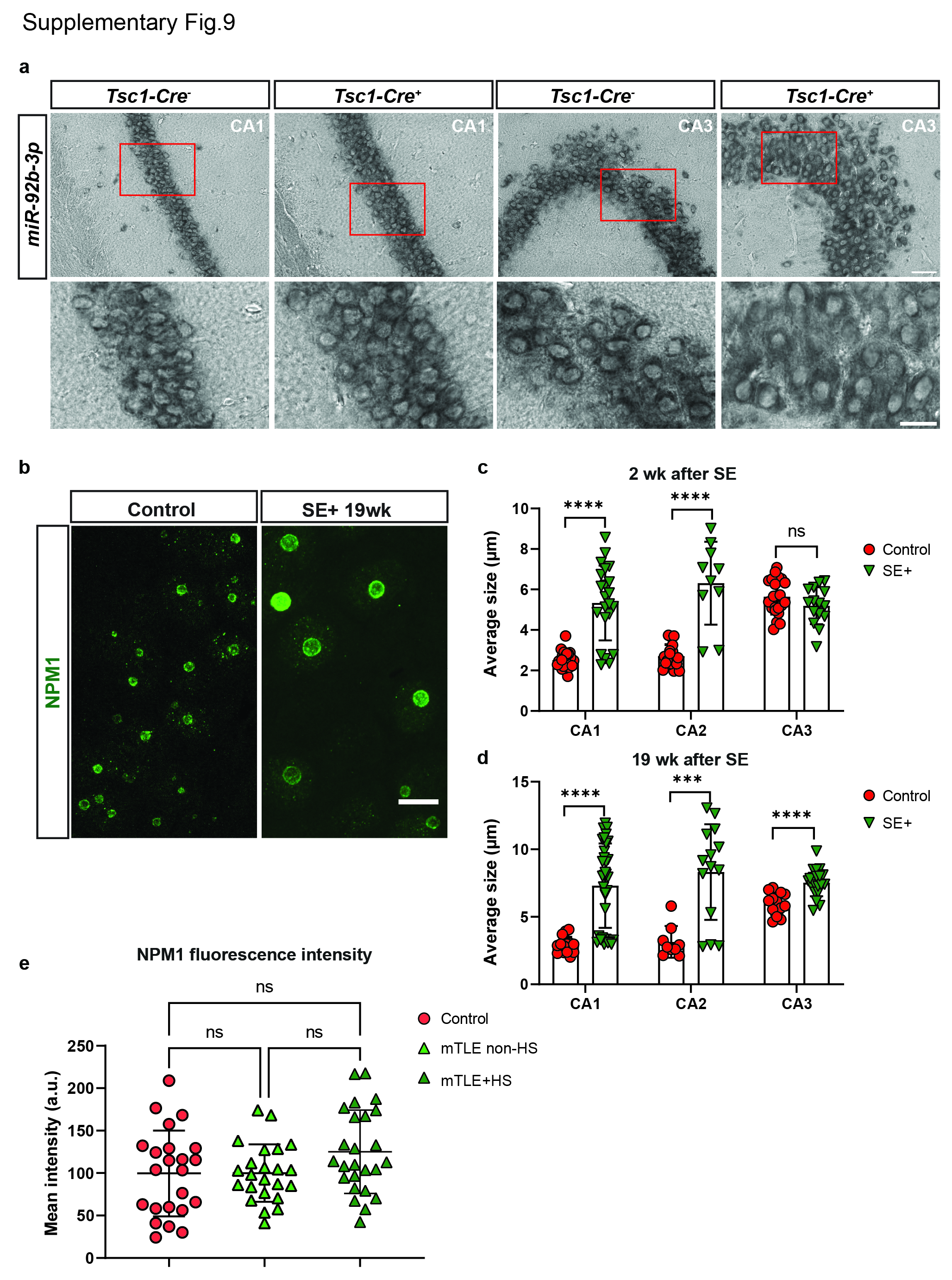

Supplement: Supplementary file 10 — Supplementary file10 (TIF 13282 kb) [file 401_2024_2817_MOESM10_ESM.tif]

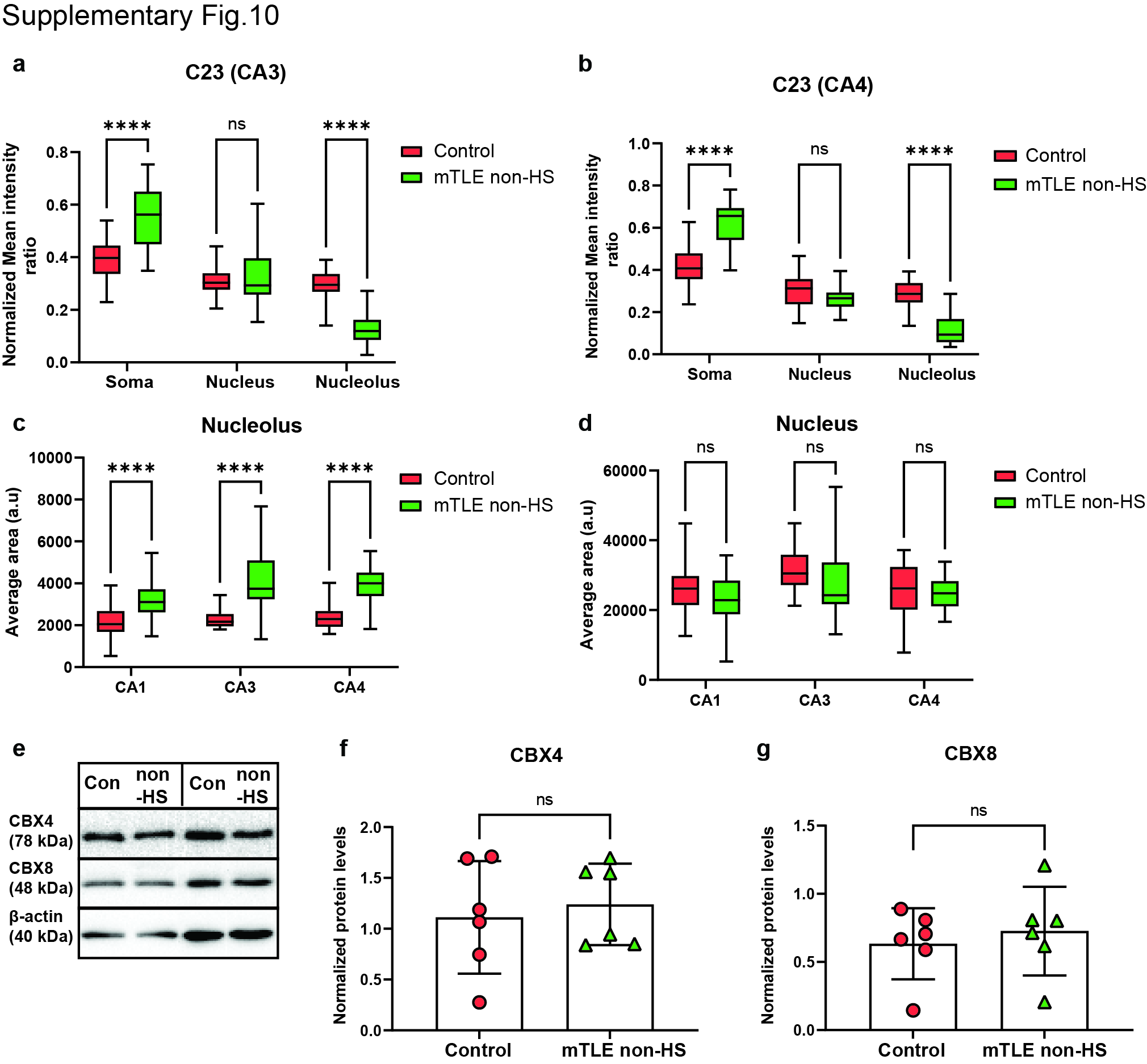

Supplement: Supplementary file 11 — Supplementary file11 (TIF 1556 kb) [file 401_2024_2817_MOESM11_ESM.tif]

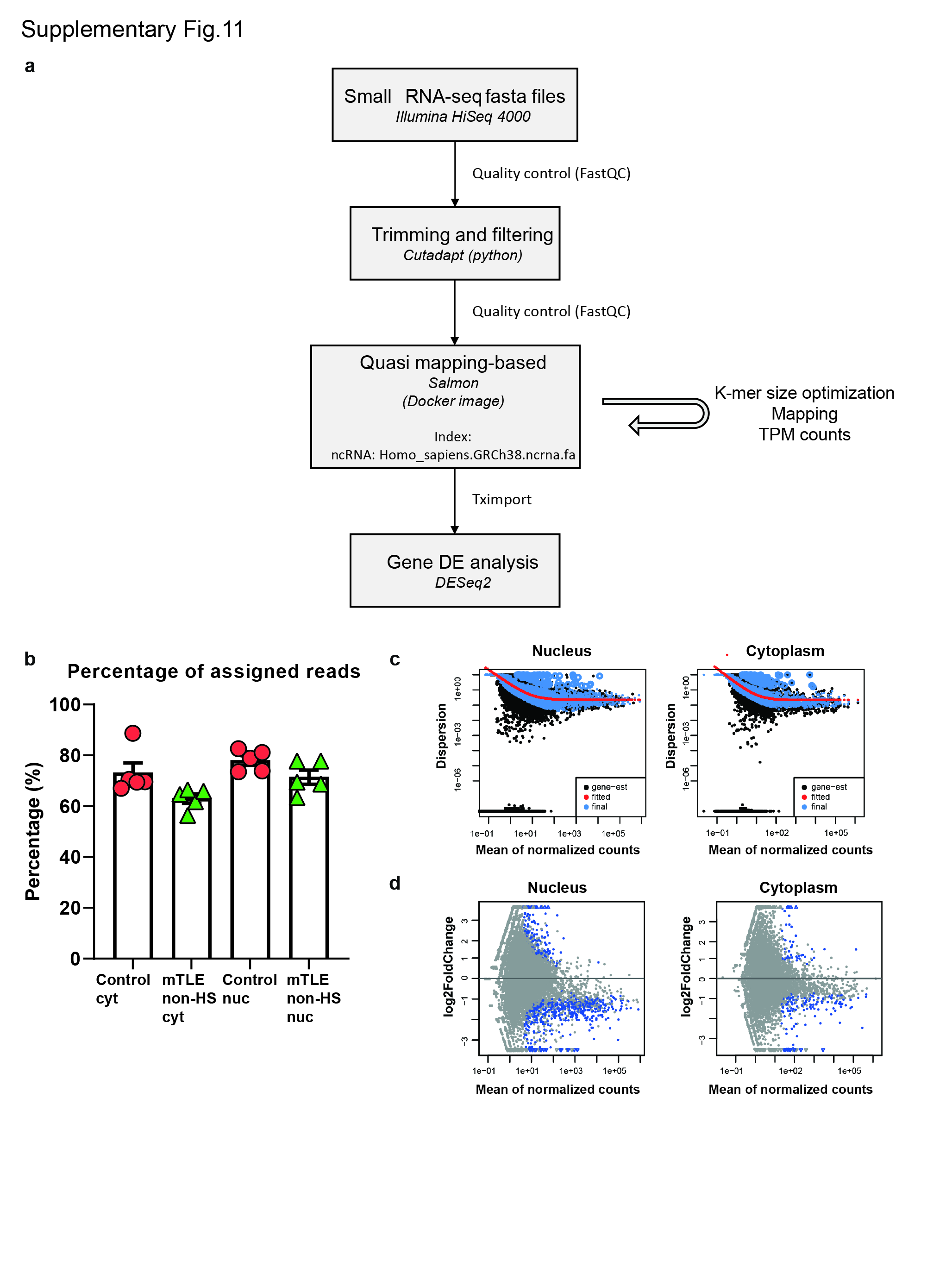

Supplement: Supplementary file 12 — Supplementary file12 (TIF 2088 kb) [file 401_2024_2817_MOESM12_ESM.tif]

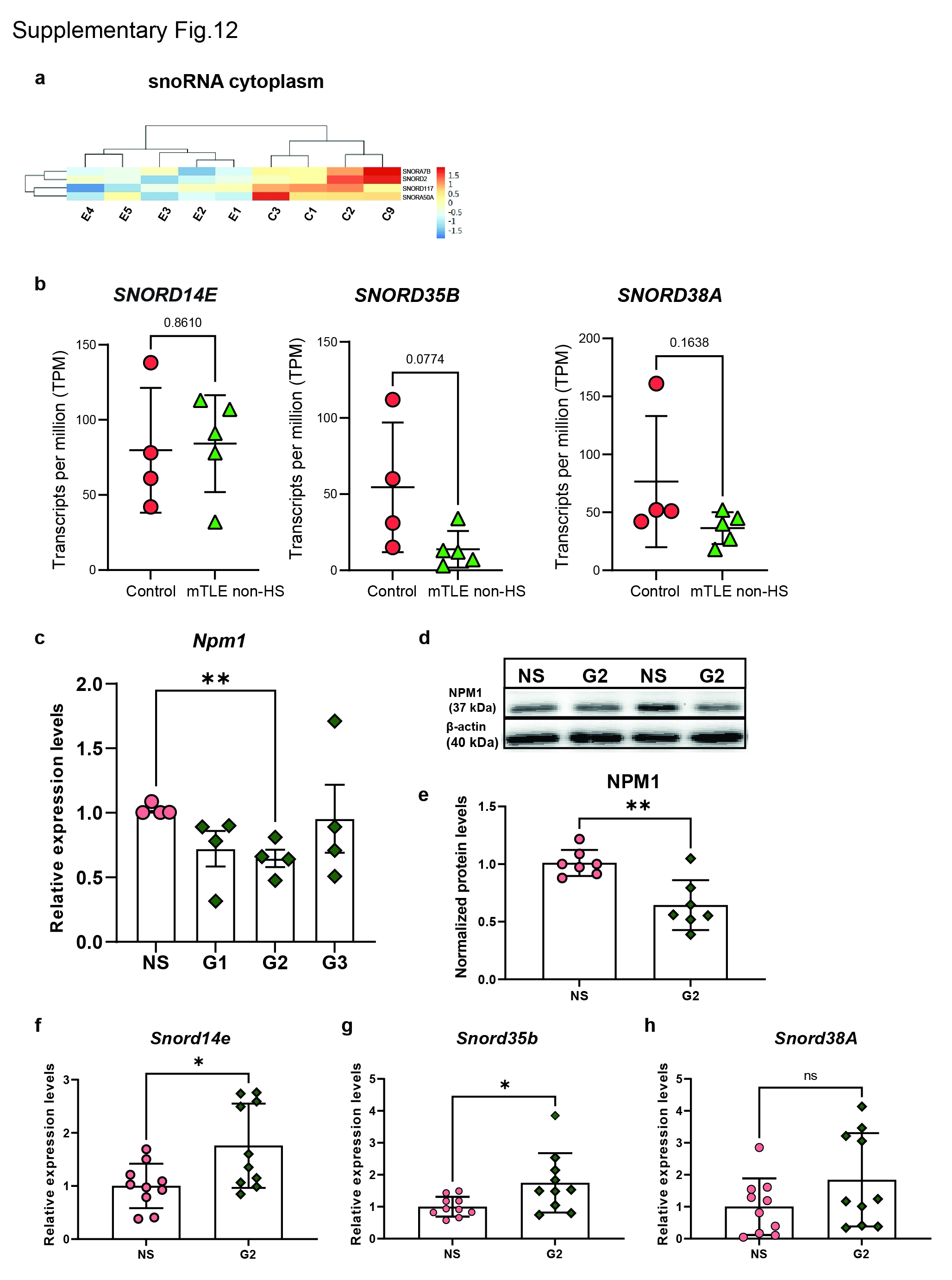

Supplement: Supplementary file 13 — Supplementary file13 (TIF 1838 kb) [file 401_2024_2817_MOESM13_ESM.tif]
